# Liposomal siRNA Formulations for the Treatment of Herpes Simplex Virus-1: In Vitro Characterization of Physicochemical Properties and Activity, and In Vivo Biodistribution and Toxicity Studies

**DOI:** 10.3390/pharmaceutics14030633

**Published:** 2022-03-13

**Authors:** Doaa Jbara-Agbaria, Saskia Blondzik, Anke Burger-Kentischer, Majd Agbaria, Mirjam M. Nordling-David, Anna Giterman, Gil Aizik, Steffen Rupp, Gershon Golomb

**Affiliations:** 1 Institute for Drug Research, School of Pharmacy, Faculty of Medicine, The Hebrew University of Jerusalem, Jerusalem 9112001, Israel; doaa.jbara@mail.huji.ac.il (D.J.-A.); majd.agbaria@mail.huji.ac.il (M.A.); mirjamnordling@hotmail.com (M.M.N.-D.); anna.fireman236@gmail.com (A.G.); gil.aizik@gmail.com (G.A.); 2 Fraunhofer Institute for Interfacial Engineering and Biotechnology, 70569 Stuttgart, Germany; saskia.blondzik@gmx.de (S.B.); anke.burger-kentischer@igb.fraunhofer.de (A.B.-K.); steffen.rupp@igb.fraunhofer.de (S.R.); 3 The Center for Nanoscience and Nanotechnology, The Hebrew University of Jerusalem, Jerusalem 9190401, Israel

**Keywords:** nanomedicine, liposomes, gene delivery, HSV-1, 3D epidermis model

## Abstract

Herpes simplex virus-1 (HSV-1) is highly contagious, and there is a need for a therapeutic means to eradicate it. We have identified an siRNA (siHSV) that knocks down gene expression of the infected cell protein 0 (ICP0), which is important in the regulation of HSV infection. The selected siHSV was encapsulated in liposomes to overcome its poor stability, increase cell permeability, and prolonging siRNA circulation time. Several siRNAs against ICP0 have been designed and identified. We examined the role of various parameters, including formulation technique, lipids composition, and ratio. An optimal liposomal siHSV formulation (Lip^DOPE^-siHSV) was characterized with desirable physiochemical properties, in terms of nano-size, low polydispersity index (PDI), neutral surface charge, high siHSV loading, spherical shape, high stability in physiologic conditions in vitro, and long-term shelf-life stability (>1 year, 4 °C). The liposomes exhibited profound internalization by human keratinocytes, no cytotoxicity in cell cultures, no detrimental effect on mice liver enzymes, and a gradual endo-lysosomal escape. Mice biodistribution studies in intact mice revealed accumulation, mainly in visceral organs but also in the trigeminal ganglion. The therapeutic potential of siHSV liposomes was demonstrated by significant antiviral activity both in the plaque reduction assay and in the 3D epidermis model, and the mechanism of action was validated by the reduction of ICP0 expression levels.

## 1. Introduction

The αHerpesvirus subfamily of the human herpesviruses causes recurring latent and lytic infections affecting ~90% of adults worldwide [1]. Herpes simplex virus-1 (HSV-1) typically causes lytic infections of the skin or mucus membranes of the mouth or lips, which are visible as fever blisters (cold sores) following epithelial cells lysis [2]. The virus can cause life-threatening complications in immunocompromised individuals. Severe complications occur when the virus infects the eyes (herpes keratitis), invades the CNS (herpes encephalitis), or is transmitted to a newborn (neonatal herpes) [3]. The gold standard of anti-HSV treatment is the guanosine analogue, acyclovir [4], which competitively inhibits and inactivates HSV-specified DNA polymerases. Although current acyclovir and other guanosine analogues inhibit viral replication, alleviate symptoms, and shorten the period of symptomatic infections, there is no prevention of recurrences and/or spreading of the herpetic infection [5]. In addition, side-effects are often underappreciated; acute kidney injury induced by acyclovir and valacyclovir has been reported at high doses [6,7,8], and resistance has become an important clinical problem as well [9,10]. Thus, there is a need for a therapeutic means eradicating HSV-1.

Herpes viruses have two distinct phases of its viral life cycle: lytic and latent; they lay dormant inside neuronal cells (sensory ganglia), evading the immune system and causing a lifelong infection [11]. The trigeminal ganglion is the main sanctuary site of the latent HSV-1 in the head and neck. Nevertheless, the virus has been detected in other sensory and autonomic ganglia, including the dorsal root, geniculate, vestibular, spiral, nodose (inferior vagal), superior cervical, and ciliary ganglia [12,13]. During latency, HSV-1 is maintained as a silenced DNA within the nuclei of sensory ganglia, where the only abundant viral gene product is a non-coding RNA, the latency-associated transcript (LAT) [14,15]. Several miRNAs are implicated in establishing latency [14,16], and one of these miRNAs, miR-H2-3p, is a transcribed antisense to infected cell protein (ICP0), a viral immediate/early protein. In the nucleus of the infected host cell, ICP0 promotes transcription from viral genes, disrupts structures in the nucleus, alters the expression of host and viral genes, and evades the host cell’s intrinsic and innate antiviral defenses. These properties of ICP0 have been suggested as possible targets for HSV-1 antiviral therapies [17,18].

siRNA therapeutics have been applied most notably in different types of cancer [19,20,21] and genetic disorders [22], and in viral infections [23]. Several studies demonstrate the therapeutic potential of siRNA for inhibiting HSV-1 and HSV-2 infection in vitro [23,24,25,26,27,28,29]. A therapeutic effect has been demonstrated in rodents following local intravaginal application of siRNAs complexed with a transfection agent [30,31], and by a topical administration of siRNA solution in a corneal infection model [32]. Nevertheless, there are neither reports on a non-viral siRNA delivery system (e.g., nanoparticles; NPs) for systemic administration nor on targeting ICP0 for HSV-1 therapy [33]. We hypothesized that treatment with an siRNA against ICP0 could provide effective therapy of HSV-1 infection. It is well known that the foremost obstacle that must be overcome before siRNAs can efficiently be used in clinical practice is the issue of delivery [34,35,36,37,38]. Topical or local treatment could serve as an administration route for eradicating the lytic virus in the skin or mucus membranes. However, to inhibit the latent virus in peripheral neurons (trigeminal ganglion), systemic delivery or a local application is required. We aimed to formulate siHSV liposomes addressing the major parameters in the rational design of an siRNA delivery system, including high siRNA loading, biological stability and extended half-life, high internalization into target cells, and endosomal escape for an efficient gene silencing [39,40,41,42,43]. We describe here proof-of-concept studies describing the design of siRNAs against ICP0; formulation approaches; liposomal siHSV physicochemical properties; cellular uptake; antiviral activity; and the mechanism of Lip-siHSV in 2D and 3D in vitro models, biodistribution, and toxicity.

## 2. Materials and Methods

### 2.1. Design of siRNA Sequences

Several miRNAs were identified in latently infected ReNcell VM, using an NGS (Next-Generation Sequencing) sequencing approach. A differentiated ReNcell VM cell line was used (Merck Millipore, Darmstadt, Germany), which develops a latent state of HSV1-infection. To characterize the early expression changes after infection, NGS analyses were carried out on differentiated ReNcell VM cells infected with HSV-1. The RNA was extracted from cells’ lysates of the individual test batches, and cDNA libraries were created and were examined by Illumina sequencing. The samples were sequenced with a reading length of 50 bp (single-end) and an average sequencing depth of approximately 89 million and 22 million reads per sample for mRNA and miRNA, respectively. The reads obtained, representing the sequenced fragments, were compared to the corresponding reference genome (HSV-1 strain 17 syn+ genome, NC_001806), yielding a quantitative expression pattern of the individual transcripts.

The selected miR-H2 sequence, 23 bp (sense strand, 5′-CCU GAG CCA GGG ACG AGU GCG CC-3′), was chosen and synthesized for comprehensive characterization (termed siHSV; IDT, Syntezza Bioscience, Jerusalem, Israel). An siRNA standard negative control (ON-TARGETplus, a non-targeting control pool; Dharmacon, Germany) and a FAM labelled siRNA (siFAM, GenePharma, Suzhou, China) were also used in this work.

### 2.2. Liposome Preparation

The liposomal formulations Lip^DSPC^, Lip^DSPE^, Lip^DOPE^, Lip^DOD2,^ and Lip^DOD3^ (Nos., 1–3, 5 and 6; Table S1) were prepared by the modified thin film hydration technique, and Lip^DOD/DOT^ (No. 4) by a modified ethanol injection technique [44,45].

#### 2.2.1. Modified Thin Film Hydration Technique

The liposomal formulations Lip^DSPC^, Lip^DSPE^, and Lip^DOPE^ (Nos., 1–3, Table S1) were composed of the positively charged lipid, DOTAP (1,2-dioleoyl-3-trimethylammonium propane; Lipoid, Ludwigshafen, Germany), and DSPC (1,2-distearoyl-*sn*-glycero-3-phosphocholine; Lipoid), DSPE (1,2-distearoyl-*sn*-glycero-3-phosphoethanolamine; Avanti Polar Lipids, Inc., Alabaster, AL, USA) or DOPE (1,2-dioleoyl-*sn*-glycero-3-phosphoethanolamine; Lipoid), cholesterol (Sigma-Aldrich, Rehovot, Israel), and DSPE-mPEG2000 (N-[Carbonyl-methoxypolyethyleneglycol-2000]-1,2-distearoyl-*sn*-glycero-3-phosphoethanolamine sodium salt; Lipoid). The lipids molar ratio was 1:3:2:0.3, 1:1:2:0.3, and 1:1:2:0.3, DOTAP:DSPC:Cholesterol:DSPE-PEG, DOTAP:DSPE:Cholesterol:DSPE-PEG and DOTAP:DOPE:Cholesterol:DSPE-PEG, respectively. The additional formulations, Lip^DOD2^ and Lip^DOD3^ (Nos. 5 and 6; Table S1), were composed of DODMA (1,2-Dioleyloxy-3-dimethylaminopropane; NOF America, New York, NY, USA), DSPC, cholesterol, and DSPE-PEG, in a molar ratio of 5:1:4:0.16 and 7.5:1:4:0.16, respectively.

The lipids were dissolved in tert-butyl alcohol and were lyophilized overnight. The lipids film was hydrated with siHSV solution (RNase free TE buffer, pH 7), in different N:P ratios (cation to anion molar ratio i.e., the cationic lipid nitrogen to siRNA’s phosphate ratio). The DOTAP:siHSV ratio in Lip^DSPC^ was 26:1 (0.25 mg/mL); Lip^DSPE^, 16:1 (0.25 mg/mL); and in the Lip^DOPE^ formulations, 16:1 or 5:1 (0.25 and 0.78 mg/mL, respectively). For Lip^DOD2^ and Lip^DOD3^, which contained DODMA, the lipids film was hydrated with siHSV solution in acidic pH 4 for efficient siRNA loading (1 mg/mL, TE buffer), in a N:P ratio of 10:1. The hydrated films of all formulations were rotated in a 60 °C bath, for 40 min at 90 rpm. The size of the obtained multilamellar vesicles was reduced by means of a Vibra-Cell tip sonicator (Sonics and Materials, Inc., Newtown, CT, USA) for 6 min, under constant voltage of 20 W, followed by filter sterilization (0.2 µm). Empty liposomes were similarly prepared omitting siHSV.

#### 2.2.2. Ethanol Injection Method

The Lip^DOD/DOT^ formulation (#4; Table S1) was prepared by the ethanol injection method (attempts to use the hydration technique resulted in a sticky layer of lipids after lyophilization rather than a film). The composition contained DOTAP, DODMA, DOPC (1,2-dioleoyl-*sn*-glycero-3-phosphocholine; Avanti), cholesterol, and DSPE-PEG, in a molar ratio of 3:5:7:4:0.8, respectively. The lipids were dissolved in ethanol (40 mg/mL) and were stirred for 5 min at 40 °C. siHSV solutions (0.15 and 0.5 mg/mL, TE buffer RNase free solution) were injected into the ethanolic lipids solution, yielding liposomes in two different N:P ratios (16:1 and 5:1; respectively). The liposomes were stirred for 20 min without heat, followed by removal of ethanol under reduced pressure using a rotary evaporator (Buchi, Flawil, Switzerland). The obtained multilamellar vesicles underwent a size reduction and filter sterilization, as described above. Empty liposomes were similarly prepared omitting siHSV.

#### 2.2.3. Fluorescently Labeled Liposomes

Fluorescently labeled liposomes (LipRhod; Liss Rhod PE 18:1 (Avanti) were similarly prepared by the modified thin film hydration technique as described above (0.125 mg/mL Rhodamine-PE in the lipids film). For determining the intracellular fate, Lip^DOPE^-siFAM was similarly prepared by replacing 5% of the siHSV with a FAM labeled siRNA (siFAM, GenePharma, Shanghai, China), using a 37.5 µM solution (based on 0.5 OD = 2.5 nmole).

#### 2.2.4. Removal of Un-Encapsulated siRNA

For removing un-encapsulated siRNA, the liposomes were dialyzed for 48 h, in excess of TE buffer (×100 of the liposomes volume) under stirring at 4 °C, using a Spectra/Por^®^ cellulose ester membrane dialysis tube (MWCO = 300 kDa, 16 × 15 mm; Spectrum Laboratories, Inc., Piscataway, NJ, USA). The buffer was exchanged during 48 h to maintain a concentration gradient. The concentration of siRNA (encapsulated and un-encapsulated) was determined by means of a NanoDrop™ 1000 Spectrophotometer (Thermo Fisher Scientific, Waltham, MA, USA).

### 2.3. Physicochemical Characterization

#### 2.3.1. Size, Surface Charge, and Morphology

Liposome size and surface charge (ζ potential) were determined by means of DLS (dynamic light scattering) at room temperature (Zetasizer Nano-ZSP, Malvern Instruments, Malvern, UK). The mean size and PDI are presented as intensity distribution. The liposome morphology was determined by cryogenic TEM (FEI Tecnai 12 G2 TWIN operated at 120 kV and equipped with a Gatan model 626 cold stage). The liposomes were first deposited on a glow-discharged TEM grid (300 mesh Cu grid) coated with a holey carbon film (lacey substrate, Ted Pella, Inc., Redding, CA, USA) and vitrified rapidly in liquid ethane using a vitrification robot system (Vitrobot mark IV, FEI, Thermo Fisher Scientific).

#### 2.3.2. siRNA Loading, Encapsulation Yield, and Lipids Content

For extracting the encapsulated siRNA, OGP (Octyl β-D-glucopyranoside; Carbosynth, UK) was used. To determine unentrapped siRNA, specimens of liposomes were centrifuged at 4000 RCF for 45 min, by means of a Vivaspin centrifugal concentrator (MWCO 300 kDa). After extensive vortex, siHSV concentration was determined by the NanoDrop method against a calibration curve, made of empty liposomes (with similar lipids concentration) spiked with different concentrations of siRNA diluted in TE buffer (in duplicates). The actual siRNA loading (µg/mL) and the encapsulation yield (%) were calculated using the following equations:

siRNA loading=Conc. of total siRNA−Conc. of unentrapped siRNA

$$\mathrm{Encapsulation}\;\mathrm{yield}\;\left(\%\right)=\left(\frac{\mathrm{siRNA}\;\mathrm{loading}}{\mathrm{Initial}\;\mathrm{siRNA}\;\mathrm{conc}.}\right)\times 100$$

The content of phospholipids in the liposomes was determined by means of HPLC (Waters, Milford, MA, USA) equipped with an ELS detector (Alltech 3300, Grace, Salt Lake City, UT USA), as described previously [46].

#### 2.3.3. Lip-siRNA Stability

Degradation and protein adsorption of Lip-siHSV were examined in physiologic conditions. siHSV-containing and empty liposomes were incubated in FBS (fetal bovine serum, Biological Industries, Israel). Following incubation at 37 °C under constant rotation (2 mg/mL liposomes, 10% *v*/*v* FBS in TE buffer), changes of size and zeta potential were determined over time. At each time point (0, 24, and 48 h), an aliquot of 10 µL was diluted to 1 mL with TE buffer, and liposomes size and zeta potential were determined by means of DLS. For evaluating siRNA integrity, free siHSV (0.46 μg/mL) and siHSV liposomes (Lip^DOPE^, 9.4 mg/mL lipids and 0.46 μg/mL siHSV) were incubated in FBS (50% *v*/*v*) for 1 and 24 h at 37 °C under constant rotation, in comparison to liposomes and free siHSV incubated in RNase free TE buffer. At the end of the incubation period, the total RNA was extracted from specimens using the mirVana™ miRNA Isolation Kit (Ambion, Thermo Fisher Scientific) according to the manufacturer’s protocol. The extracted siHSV concentration was determined by means of a NanoDrop™ 1000 Spectrophotometer (Thermo Fisher Scientific). RNA samples were thereafter loaded onto a 24% native polyacrylamide gel, and electrophoresis was carried out at 200 V for 1.5 h, in Tris-Borate-EDTA (TBE) as the running buffer. siRNA bands were stained using SYBER^®^ Gold Nucleic Acid Gel Stain (1:1000 *v*/*v*, InvitrogenTM, USA) and visualized under a UV transilluminator (ChemiDoc XRS+ System; BIO-RAD, Hercules, CA, USA).

The shelf-life stability of Lip-siRNA, suspended in TE buffer and stored at 4 °C/dark, was determined by analyzing the changes in terms of size, PDI, and zeta potential, over a period of 1.5 year by means of DLS (Zetasizer Nano-ZSP, Malvern Instruments, Malvern, UK).

### 2.4. Cell Culture Studies

Spontaneously immortalized human keratinocyte cell line (HaCaT; CLS Cell Lines Service, Eppelheim, Germany) was routinely cultivated in Dulbecco’s modified Eagle medium (DMEM) supplemented with 10% FBS, 2 mM L-glutamine, and 100 units/mL penicillin and 100 mg/mL streptomycin (Biological Industries), at 37 °C and in a humidified 5% CO_2_ atmosphere. Primary smooth muscle cells (SMC) were isolated from a rat’s aorta (Sabra; Harlan Laboratories, Jerusalem, Israel) weighing 200–300 g, as previously described [47].

#### 2.4.1. Qualitative Internalization of Liposomes

HaCaT cells were seeded on coverslips in 12-well plates (100 k cells/well), containing the complete growth medium, and were left overnight to adhere. The cells were treated on the following day with different concentrations of fluorescently-labeled empty liposomes (Lip-Rhod). The cells were washed 3 times with phosphate buffered saline (PBS), at different time points, to remove excess treatment and were fixed on coverslips with 4% formaldehyde solution for 10 min, followed by 3 more washes with PBS. The cell nuclei were stained with 10 μg/mL Hoechst 33,258 reagent solution (Sigma-Aldrich) for 10 min and mounted upside down onto microscope slides. Slides were analyzed by means of an Olympus FV 10i confocal laser scanning microscope (1 × 60, Olympus America, Inc., Westborough, MA, USA), followed by image analysis (Olympus FV10 ASW 4.2 viewer software).

#### 2.4.2. Quantitative Internalization of Liposomes

HaCaT cells were seeded in 24-well plates (70 k cells/well) containing the complete growth medium and were left overnight to adhere. The cells were treated on the following day with empty fluorescent liposomes at different concentrations (suspended in complete growth medium). At specified time points, the cells were washed ×3 with PBS, harvested and analyzed by means of FACS, (BDTM LSR II, BD Biosciences, Franklin Lakes, NJ, USA) for Rhodamine-PE—positive cells. Untreated cells and cells treated with unlabeled liposomes were used as controls. The number of stained cells and the fluorescence intensities were calculated based on the FACS histograms obtained (FCS Express 4 software; De Novo software, Pasadena, CA, USA).

#### 2.4.3. Intracellular Fate

HaCaT cells were seeded on coverslips in 12-well plates (100 k cells/well) containing the complete growth medium and were left overnight to adhere. Cells were incubated for 4, 24, and 48 h with fluorescently labeled siFAM liposomes (6 µg/mL total siRNAs). Late endosomes/lysosomes were stained by adding LysoTracker (75 nM, Red DND99, Life Technologies, Thermo Fisher Scientific) 1 h prior to the end of the incubation period. The cells were thereafter washed with PBS (×3), were fixed using 4% formaldehyde solution for 10 min, were washed again with PBS (×3), and were mounted onto microscope slides. Slides were analyzed by means of confocal microscopy (magnification of ×60). Untreated cells served as control.

#### 2.4.4. siHSV Stability following Internalization by HaCaT Cells

The fate of the siRNA and its stability was examined in HaCaT cells. The cells were seeded in 6-well plates (500 k cells/well) containing the complete growth medium. The cells were treated on the following day with Lip-siHSV (200 nM) in comparison to empty liposomes spiked with siHSV (200 nM). Collection and lysis of the cells, at specified time points, were carried out by the Tri reagent (Sigma-Aldrich) followed by RNA extraction according to the manufacturer’s protocol. The extracted RNA was quantified by means of NanoDrop. To evaluate siRNA integrity, equal amount of extracted RNA from each sample was examined by electrophoresis as above, and the obtained bands were semi-quantified by image analysis (ImageJ; NIH).

#### 2.4.5. In Vitro Cytotoxicity

The cytotoxicity of liposomal siHSV to SMC and HaCaT cells was examined in comparison to empty liposomes, and polyethyleneimine (PEI) solution, 50 µg/mL, served as a positive control (Sigma-Aldrich). The cells were seeded in 24- and 96-well plates, 25 k and 10 k cells/well, respectively, containing a complete growth medium. On the following day, the cells were treated with Lip-siHSV (N:P of 5:1) and empty liposomes. Cells’ viability was determined by means of the MTT assay (Thiazolyl Blue Tetrazolium Bromide, Sigma-Aldrich). The plates were quantified by means of a plate reader at λ = 540 nm, normalized to untreated cells.

### 2.5. Antiviral Activity

#### 2.5.1. Plaque Reduction Assay

HaCaT cells were seeded in 24-well plates (30 k cells/well) containing the complete growth medium. Uninfected cells and infected untreated cells served as controls. The cells were pre-treated with Lip-siHSV (N:P ratio of 5:1; 1.5, 2.5, and 6.0 µg/mL siHSV concentration), in comparison to treatments with free siHSV and scrambled siRNA (1.5, 2.5, and 6.0 µg/mL, transfected by lipofectamine RNAiMAX reagent, Invitrogen), empty liposomes, and acyclovir (125 µM). After 48 h incubation, the cells were infected with HSV-1 (Human Herpes virus, strain HF, ATCC, VR-260; 1 × 10^4^ plaque forming units (PFU) per well; 5800 PFU/cm^2^), and were fixated and stained 16 h later by means of immunohistochemistry using rabbit polyclonal anti-HSV1 (BioGenex, CA, USA). The cells were fixed with 4% paraformaldehyde, incubated with 3% H_2_O_2_, and blocked with BSA blocking solution (0.01 g/mL). The anti-HSV antibody was added at a dilution of 1:200, IgG biotin (1:100 dilution; BioGenex) was added as multi-link solution, streptavidin POD (1:100 dilution; BioGenex) was added as a label solution, and chromogen AEC (one drop for 2.5 mL Chromogen buffer; BioGenex) was incubated until a brownish red staining was visible. TBST (Tris-buffered saline, 0.1% Tween 20) was used as a washing buffer. Images were recorded by means of an Olympus Optical (IX81) microscope and an Olympus (DP25) camera.

#### 2.5.2. 3D Epidermis Model

The 3D epidermis model was used to evaluate the anti-viral effect on HSV-1 infection, as described previously [46]. Human immortalized keratinocytes (HaCaT; 50 k) were seeded on cell culture inserts (0.4 µm pore size) in 24-well plates. Cells were incubated submerged in DMEM supplemented with 10% FBS. Following 2 days of incubation, the inserts were transferred to 6-well plates and were grown at the air–liquid interface, in Keratinocyte Growth Medium 2 (KGM2) supplemented with SupplementMix (PromCell, Heidelberg, Germany) with additional 1.88 mM Ca^+2^, without both bovine pituitary extract (BPE) and human epidermal growth factor (hEGF), spontaneously differentiating to a well stratified epidermis. The inside of the insert was kept dry, and the airlift phase lasted for 5 days. The 3D tissue equivalents were pre-treated with Lip-siHSV (N:P ratio of 5:1, 2.5 µg/mL siHSV concentration), in comparison to empty liposomes and scrambled siRNA (1.5 µg/mL; with lipofectamine) as negative controls, to free siHSV (1.5 µg/mL; with lipofectamine) as a positive control, and to uninfected cells and infected untreated cells. After 48 h, the tissues were infected with HSV-1 (2.7 × 10^3^ PFU per skin tissue; 5800 PFU/cm^2^). Following 16 h of infection, the epidermis tissues were fixated using Boins solution (Carl Roth GmbH, Germany), were embedded in paraffin, and were cut into 4 µm thick sections. The tissue sections were then stained with hematoxylin (Merck KGaA, Darmstadt, Germany) and eosin B (Sigma-Aldrich), or were immunohistochemistry stained with anti HSV-1 polyclonal antibody (BioGenex) for visualization and quantification.

#### 2.5.3. ICP0 Expression Levels

HaCaT cells were seeded in 6-well plates (150 k cells/well) containing the complete growth medium. Uninfected cells and infected untreated cells served as control groups. The cells were pre-treated with Lip-siHSV (2.5 and 6.0 µg/mL siHSV concentration), in comparison to treatments with free siHSV and scrambled siRNA (0.5 and 1.5 µg/mL; transfected by lipofectamine), and empty liposomes. After 48 h incubation of the pre-treated cells, the cells were infected with HSV-1 (5 × 10^4^ PFU) for 16 h and lysed in RIPA-buffer. Cell lysates were supplemented with sample buffer (Bolt LDS Sample Buffer (4×) Thermo Fisher Scientific), heated to 70 °C, separated in a Bolt™ 4–12% Bis-Tris Plus Gel (Thermo Fisher Scientific), and transferred onto a nitrocellulose membrane with an iBlot 2 Gel Transfer Device. After blocking with 5% (*w*/*v*) nonfat milk powder in TBST, membranes were incubated with primary antibodies, α-tubulin (DM1A at 1:1000 in TBST, Calbiochem CP06) and HSV1 ICP0 (5H7 at 1:5000 in TBST, Abcam ab6513). Bound antibodies were detected with the Peroxidase AffiniPure Goat Anti-Mouse IgG antibody (1:10,000 in TBST, Jackson Immunoresearch Laboratories, Inc., West Grove, PA, USA). For detection, SuperSignal West Dura Extended Duration Substrate (Thermo Fisher Scientific) was added to the membranes, and the signals were recorded by means of a Luminescent Image Analyzer (LAS-1000 plus; Fujifilm, Tokyo, Japan).

### 2.6. Biodistribution and Toxicity In Vivo

#### 2.6.1. Animal Care

Animal care and procedures conformed to the standards for care and use of laboratory animals of the Hebrew University of Jerusalem, Israel, and the National Institute of Health (NIH, USA; ethics approval number, MD-17-15238-5). Animals were fed with standard laboratory chow and tap water ad libitum. Sixty-four female Balb/c mice (Harlan Laboratories, Israel) were used in this study.

#### 2.6.2. Biodistribution

The biodistribution of Lip-siHSV was examined in intact mice. Female Balb/c mice (Harlan Laboratories, Israel) weighing 20–22 g were randomly assigned to treatment and control groups. Fluorescently labeled Lip^DOPE^ (LipRhod) suspended in TE buffer (36 mg/mL) were IP injected (200 µL). The mice were euthanized after 4, 24, and 48 h (*n* = 4 in each group), perfused with saline, and the liver, spleen, kidneys, lungs, heart, brain, spinal cord, and trigeminal ganglia were harvested. The accumulation of the liposomes in the organs was assessed by means of fluorescent imaging (Typhoon FLA 9500, GE Healthcare, UK) followed by image analysis (ImageJ). The mean fluorescence intensity in each organ was normalized to an un-treated control (organs autofluorescence). For evaluating siRNA biodistribution by liposomes (Lip^DOPE^-siRNA) in comparison to siRNA complexed with lipofectamine, fluorescently labeled siRNA (siFAM) was co-encapsulated with siHSV (5%). Female Balb/c mice (Harlan Laboratories, Israel), weighing 20–22 g, were randomly assigned to treatment and control groups and were treated with IP injections (2 mg/kg). The mice were euthanized after 24 h (*n* = 4 in each group), perfused with saline, and the liver, spleen, kidneys, lungs, and heart were harvested. The mean fluorescence intensities were assessed as detailed above.

#### 2.6.3. Lip-siHSV Toxicity

The possible hepatotoxicity of Lip-siHSV toxicity was examined following IV injection of liposomes to female Balb/c mice (Harlan Laboratories, Israel). Thirty-six mice weighing 22–23 g were randomly assigned to treatment and control groups and were divided into two time points (24 h and 7 days). In the treatment groups, mice were IV injected with Lip^DSPC^-siRNA at a dose of 2 μg/kg body-weight of siRNA (212 mg/kg lipids; *n* = 8). In the control groups, mice were IV injected with empty non-PEGylated positively charged Lip^DSPC^ (233 mg/kg lipids, *n* = 8), empty negatively-charged liposomes (367.5 mg/kg, *n* = 8), saline (*n* = 8), or LPS (*E. coli* lipopolysaccharides; *n* = 4) at a dose of 1 mg/kg (IP). Half of each group was sacrificed after 24 h and the other half after 7 days. Blood was collected (heparinized syringe) via cardiac puncture under general anesthesia, and the plasma was separated by centrifugation at 4000 rpm for 10 min. Plasma levels of alanine transaminase (ALT) and aspartate aminotransferase (AST), enzymes typically used as biomarkers for hepatic damage and toxicity, were determined according to the routine protocol of the Department of Clinical Biochemistry, Hadassah Hospital, Jerusalem, Israel.

### 2.7. Statistical Analysis

All data are expressed as the mean ± standard deviation unless noted otherwise. For statistical analysis, the one-way analysis of variance (ANOVA) with Tukey’s post hoc analysis was used. Differences were considered significant at *p* < 0.05.

## 3. Results

### 3.1. siHSV Identification

**Figure 1:**
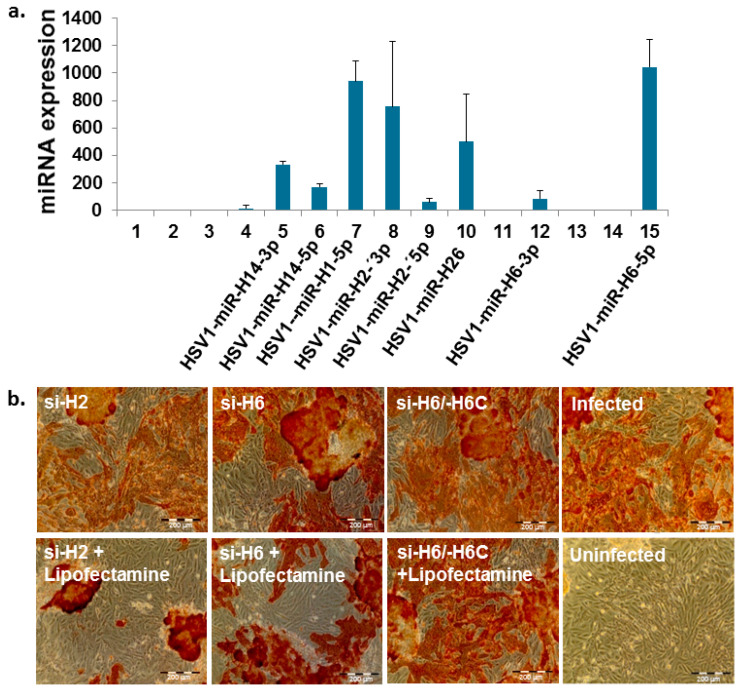
(**a**) Identification of potential microRNAs in the latent state of HSV-1 infection against HSV-1 target protein, ICP0, by means of NGS-sequencing of differentiated, latently infected human neural progenitor cell line (ReNcell VM). A set of viral miRNAs highly expressed in differentiated ReNcell VM cells has been identified, and the miRNAs sequences, miR-H2 and miR-H6, have been further analyzed for antiviral activity; (**b**) the antiviral effect of the respective siRNAs in the plaque reduction assay (a confluent layer of HaCaT cells): free si-H2 and siH6 (upper panel) in comparison to siRNAs transfected by lipofectamine (lower panel). The formed syncytia, shown in brownish red, was visualized following immunohistochemistry staining of the cells (anti-HSV1 antibody). Brownish red depicts the envelope-protein of the virus, indicating proliferative infection (scale bar = 200 μm).

The screening of miRNAs in the latent state of HSV1-infected ReNcell VM cells, using NGS (see Materials and Methods), yielded a set of highly expressed microRNAs (Figure 1a), and several of them have been reported to affect expression of the HSV-1 target protein, ICP0. This includes two miRNAs sequences, miR-H2 and miR-H6 (Figure 1a), that have been shown to be derived from LAT [14]. The sequence of miR-H2 and miR-H6 [14], confirmed by NGS in our analysis, was used to design the respective siRNAs si-H2 and si-H6. si-H2 (termed, “siHSV”) was found most potent against HSV-1 infection in a plaque reduction assay (Figure 1b) and was selected for further studies. The location of the miR-H2 and miR-H6 on the HSV-1 genome is described in (Figure S1).

### 3.2. siHSV Liposomes

Various PEGylated liposomal formulations encapsulating siHSV have been designed, varying in the formulation technique, lipid composition (different lipids and counter-ion compositions: cationic lipids, ionizable cationic lipids, helper lipids, and PEGylated lipids), and the ratio between the cationic lipids and siHSV. The various lipids examined were DOTAP and DODMA (permanent and ionizable cationic lipids; respectively) for siHSV encapsulation and enhancing endosomal escape [48], DOPE (endosomal escape helper lipid), DOPC (neutral helper lipid) [49], DSPC and DSPE (structural lipids) [50], DSPE-PEG (for PEGylation) [51], and cholesterol (for structural stability) [52,53]. The six optimal liposomal formulations (Table S1) were chosen based on particle size (100–200 nm), PDI (<0.2), and siHSV loading (>50 µg/mL). Results obtained for the best formulation, Lip^DOPE^-siHSV, are described below. Description of all the other formulations, including cellular uptake, cytotoxicity, and antiviral activity, is detailed in the Supplementary Data.

#### 3.2.1. Lip^DOPE^-siHSV Physicochemical Properties

**Figure 2:**
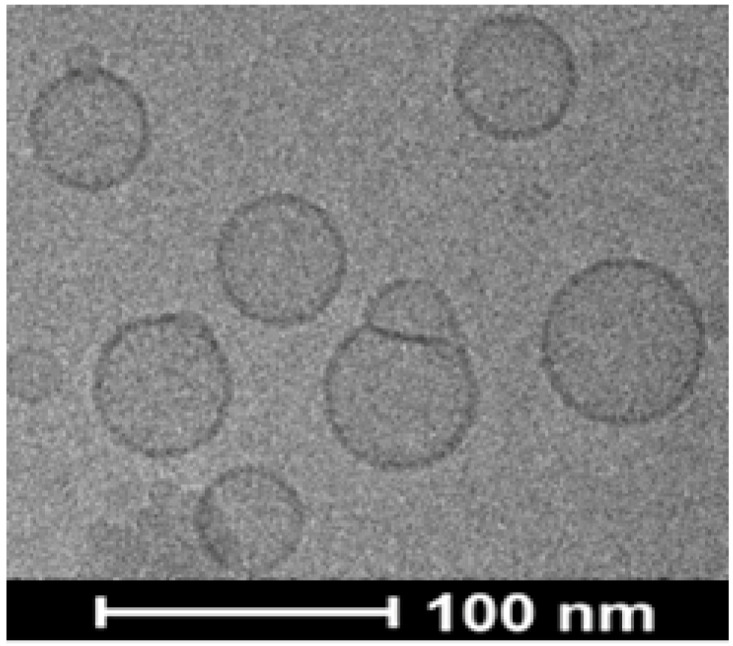
A representative cryo-TEM image depicting the structure of Lip^DOPE^-siHSV (N:P ratio 5:1) (scale bar = 100 nm).

The best formulation, Lip^DOPE^ (#3 in Table S1), was prepared in two different DOTAP:siHSV ratios (N:P, 16:1 and 5:1; Table 1). Both formulations were characterized with desirable physicochemical properties in terms of high siHSV loading (214 ± 25 and 670 ± 36 µg/mL, respectively), near-neutral surface charge (2.7 ± 0.1 and 3.3 ± 0.2 mV, respectively), and in a nano-range size (142 ± 1 and 133 ± 2 nm, respectively) with low PDI (0.21 ± 0.03 and 0.19 ± 0.02, respectively). In addition, cryo-TEM images revealed homogeneous population of unilamellar and spherical-shaped vesicles (Figure 2). Since Lip^DOPE^-siHSV with N:P ratio of 5:1 resulted in higher siHSV loading, it was selected for further studies.

#### 3.2.2. Stability

**Figure 3:**
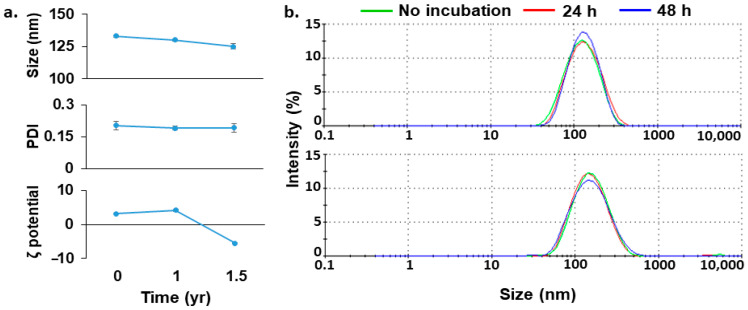
Stability and shelf-life of Lip^DOPE^-siHSV. (**a**) The long-term shelf-life stability of Lip^DOPE^-siHSV, suspended in TE buffer and stored at 4 °C, in terms of size, PDI, and surface charge changes. (**b**) The structural stability of Lip^DOPE^-siHSV (bottom) over time in comparison to empty liposomes (top) following incubation in 10% *w*/*v* bovine serum albumin at 37 °C.

Lip^DOPE^-siHSV, suspended in TE buffer and stored at 4 °C for 1.5 yr., was found stable, as evidenced from the insignificant changes of size, PDI, and surface charge (Figure 3a). In addition, siHSV liposomes incubated in serum were found stable after 24 and 48 h, as suggested from the insignificant change of size (Figure 3b).

**Figure 4:**
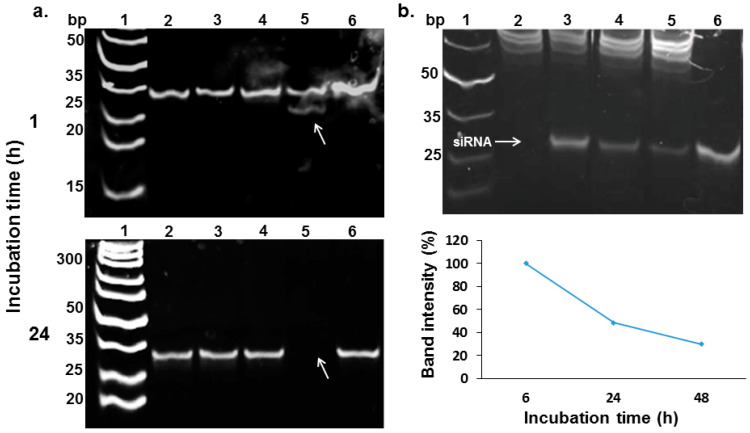
Evaluation of encapsulated siHSV stability in simulated physiological conditions. (**a**) The integrity of siHSV extracted from Lip-siHSV following incubation in 50% bovine serum albumin at 37 °C for 1 and 24 h: (1) Ladder; (2) extracted siHSV incubated in TE buffer; (3) extracted siHSV incubated in serum; (4) free siHSV incubated in TE buffer; (5) free siHSV incubated in serum; and (6) free siHSV without incubation. An aliquot of each sample (200 nM) was analyzed by native 24% acrylamide gel, stained with SYBER Gold. (**b**) The integrity of encapsulated siHSV in comparison to a spiked sample of empty liposomes with siHSV (200 nM) in keratinocytes (HaCaT) cell cultures: (1) ladder; (2) empty liposomes spiked with siHSV after 6 h; (3) Lip-siHSV after 6 h; (4) Lip-siHSV after 24 h; (5) Lip-siHSV after 48 h; and (6) free siHSV with no incubation. The semi-quantification (ImageJ analysis) of siHSV bands intensities is shown at the lower right-hand part of b. An aliquot from each sample of extracted RNA was analyzed (200 nM) by native 24% acrylamide gel, stained with ethidium bromide, and visualized. Free siHSV with no incubation (lane 6) was set as 100% recovery.

Gel electrophoresis studies revealed that siHSV extracted from liposomes remained intact, neither damaged by the preparation process nor following incubation in serum (Figure 4a). In addition, siHSV remained intact in keratinocytes (HaCaT) cell culture following internalization of the liposomal formulation in comparison to empty liposomes spiked with siHSV (Figure 4b).

### 3.3. Cellular Uptake

**Figure 5:**
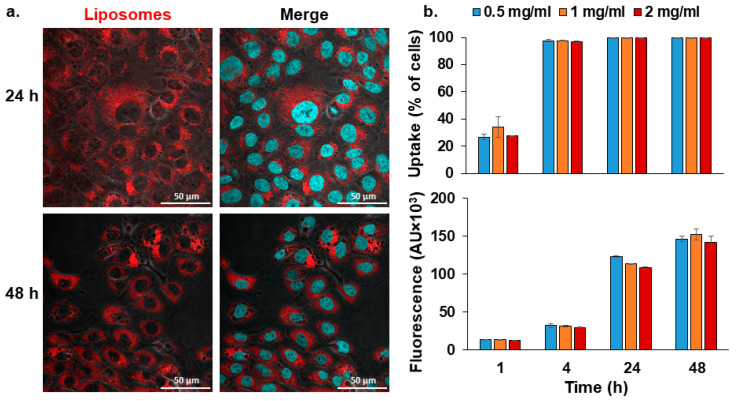
The internalization of Lip^DOPE^ by keratinocytes (HaCaT) cell culture. Qualitative (confocal microscopy; (**a**) quantitative (flow cytometry analysis; and (**b**) assessment of cellular uptake of Lip^DOPE^ (fluorescently labeled with Rhodamine-PE; 2 mg/mL lipids concentration). The liposomal membrane is depicted in red, and cell’s nuclei in cyan (scale bar = 50 μm). The fluorescence intensities were normalized to untreated cells (mean ± SD).

The internalization of Lip^DOPE^ by keratinocytes was examined by incubating HaCaT cells with fluorescently labeled liposomes (Rhodamine-PE), followed by qualitative (confocal microscopy) and quantitative (flow cytometry) analyses (Figure 5). The cells demonstrated time-dependent but not dose-dependent internalization, exhibiting profound internalization after 24 and 48 h, and virtually all cells were stained following 4 h of treatment. Similar results have been obtained for the other PEGylated formulations (Table S1) varying in terms of formulation technique and lipid composition (Figures S3–S5).

### 3.4. Cytotoxicity

**Figure 6:**
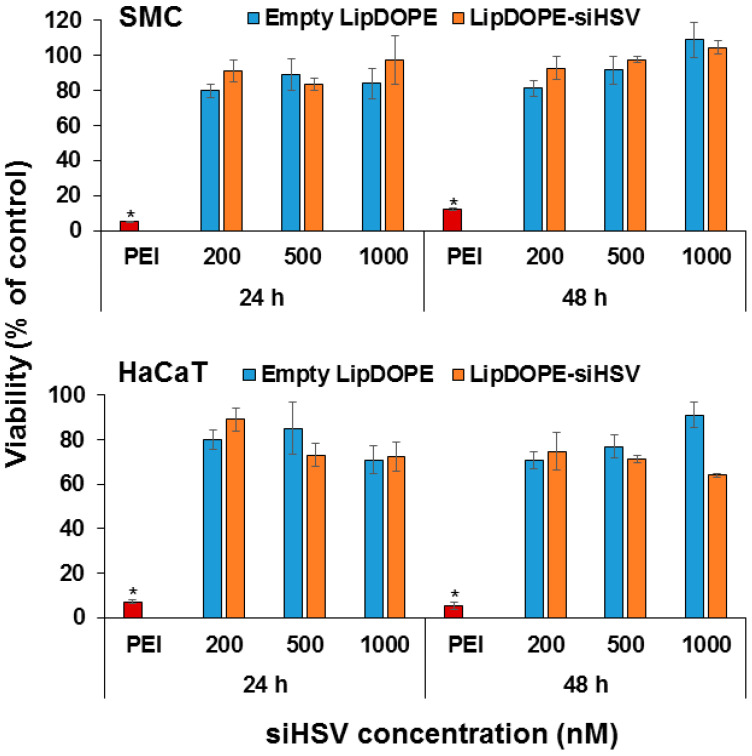
The dose- and time-dependent cytotoxicity of Lip^DOPE^ -siHSV in rat primary aortic smooth muscle cells (SMC) and keratinocytes (HaCaT) cell cultures. The cytotoxicity was determined by means of the MTT assay, and cell viability was normalized to untreated cells (*n* = 4; * *p* < 0.01).

The cytotoxicity of Lip^DOPE^ was evaluated in SMC and HaCaT cultures (Figure 6). Both empty Lip^DOPE^ and Lip^DOPE^-siHSV exhibited no cytotoxic effect on SMC up to 48 h in comparison to both untreated cells and cells treated with PEI (negative and positive controls, respectively). However, moderate cytotoxicity was detected in HaCaT cells 48 h following treatment with Lip^DOPE^-siHSV at a high concentration of 1000 nM (Figure 6). Only two of the six examined formulations (formulations #4 and #6; Table S1) demonstrated cytotoxic effect in HaCaT cells (Figures S5 and S7).

### 3.5. Antiviral Effect of Lip^DOPE^-siHSV

#### 3.5.1. Antiviral Effect in the Plaque Reduction Assay

**Figure 7:**
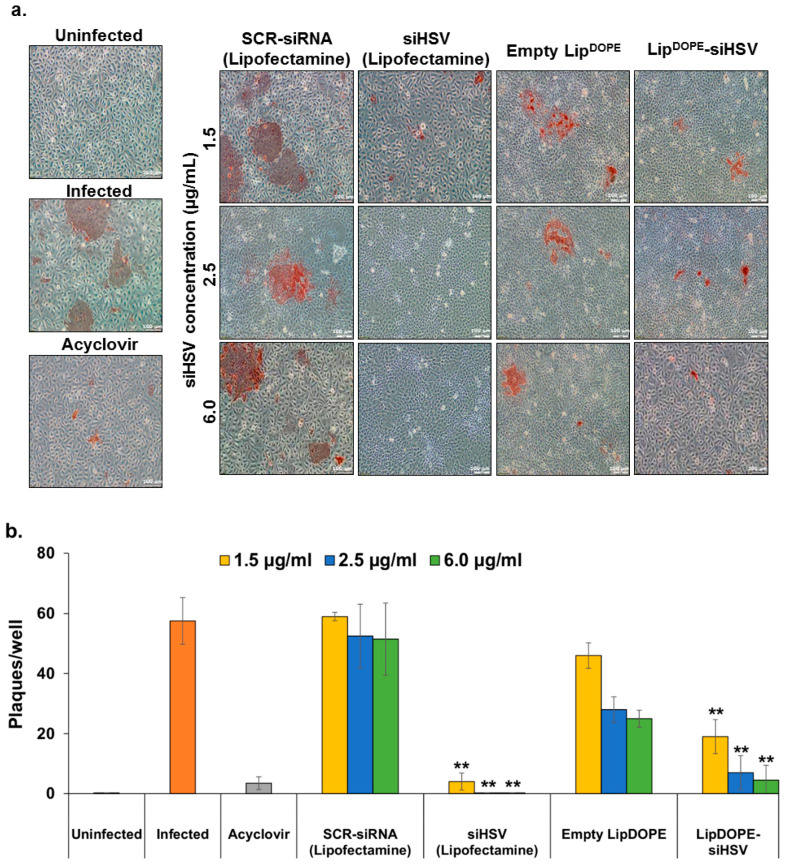
The antiviral effect of Lip^DOPE^-siHSV following treatment of HaCaT cells (keratinocytes) infected with HSV-1 (plaque reduction assay). (**a**) Treatment groups: Lip^DOPE^-siHSV, empty Lip^DOPE^ and scrambled-siRNA (SCR-siRNA transfected by lipofectamine) served as negative controls, and acyclovir (125µM) and siHSV (transfected by lipofectamine) served as positive controls, in comparison to uninfected cells and untreated infected cells. Following 48 h of treatment, the cells were infected with HSV-1 (1 × 10^4^ PFU) for 16 h. The formed syncytia, shown in brownish red, was visualized following immunohistochemistry staining of the cells (anti-HSV1 antibody; *n* = 8). Brownish red staining depicts the envelope-protein of the virus, indicating proliferative infection, and no staining indicates full protection (magnification ×10; scale bar = 100 μm). (**b**) Semi-quantitative analysis of the antiviral efficacy (** *p* < 0.01).

The dose-dependent treatment efficacy against HSV-1 infection was evaluated in a plaque reduction assay of keratinocytes. The virus syncytia—multinucleated cells formed by multiple cell fusions induced by HSV-1—were qualitatively and quantitatively determined (Figure 7). To determine specific and unspecific effects, we used siHSV and SCR-siRNA, respectively, transfected with lipofectamine, which is a well-known transfection reagent. Both treatments of Lip^DOPE^-siHSV and free siHSV (lipofectamine) exhibited significantly high antiviral efficacy similar to acyclovir treatment. In contrast, the other examined formulations (Table S1) were found to be inactive (Figures S8–S10). Lip^DOPE^-siHSV treatment resulted in dose-dependent efficacy, eradicating the virus at 2.5 and 6.0 µg/mL, whereas siHSV in lipofectamine was highly effective at a lower concentration of 1.5 µg/mL. In addition, treatments with empty liposomes resulted in modest antiviral efficacy, whereas SCR-siRNA (lipofectamine) treatment was found to be ineffective.

#### 3.5.2. Antiviral Effect in the 3D Epidermis Model

**Figure 8:**
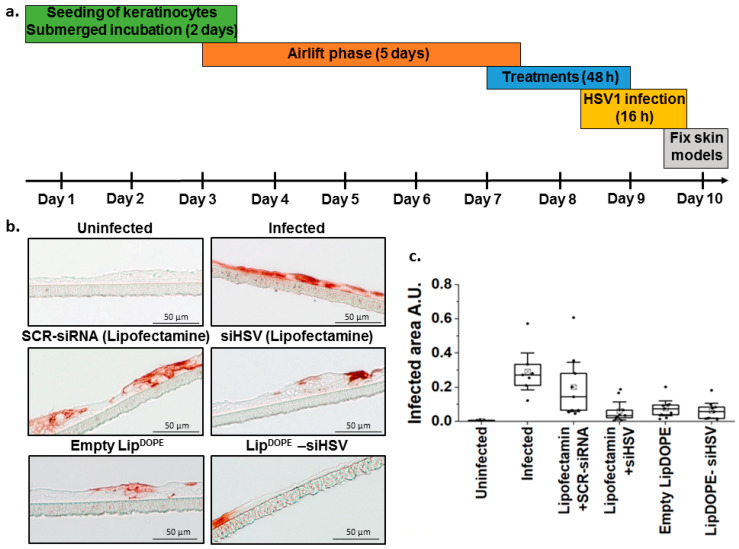
The antiviral effect of Lip^DOPE^-siHSV (DOTAP:siHSV N:P of 5:1) against HSV-1 infection in the 3D epidermis model of immortalized keratinocytes (HaCaT). (**a**) Illustration of the experimental setup. (**b**) Qualitative assessment of the treatments by immunohistochemistry of epidermis tissues treated with Lip^DOPE^-siHSV (2.5 µg/mL), scrambled-siRNA (SCR) transfected by lipofectamine (1.5 µg/mL) and empty Lip^DOPE^ (negative controls), and siHSV transfected by lipofectamine (1.5 µg/mL; positive control), in comparison to uninfected and untreated infected cells. The epidermis tissues were infected with HSV-1 after 48 h and were analyzed after 16 h for tissue morphometry and integrity (scale bar = 50 μm). (**c**) Quantification of the immunohistochemistry staining of three independent experiments (each experiment consisting of two or three individual skin models) is shown in (**b**) by calculating the infected area in the epidermis tissue.

In the 3D epidermis model generated from immortalized keratinocytes (HaCaT; see study scheme in Figure 8a), the antiviral efficacy was assessed by immunohistochemistry staining of the virus envelope-protein in histological sections (Figure 8b,c; Tables S2 and S3). Lip^DOPE^-siHSV treatment was found to be highly effective in eradicating the virus, similar to siHSV (lipofectamine) and empty liposomes, in comparison to the negative control groups, untreated infected cells, and SCR-siRNA (lipofectamine).

#### 3.5.3. ICP0 Expression Level

**Figure 9:**
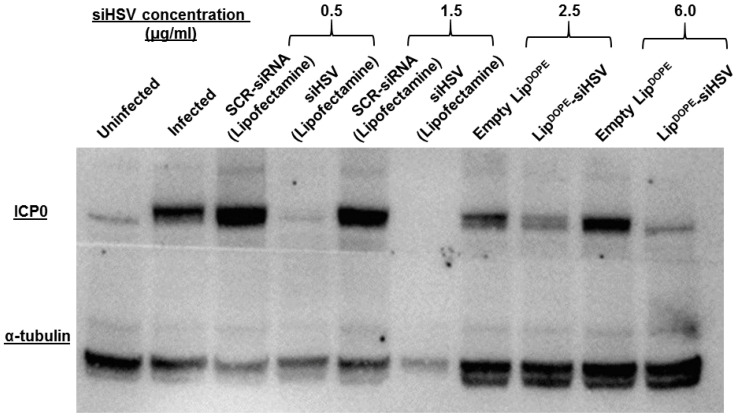
The expression levels of ICP0, following pre-treatment of HaCaT cells (keratinocytes) infected with HSV-1 (Western blot). Treatment groups: Lip^DOPE^-siHSV, empty Lip^DOPE^, and scrambled-siRNA (SCR-siRNA transfected by lipofectamine) served as negative controls, and siHSV (transfected by lipofectamine) served as positive control, in comparison to uninfected cells and untreated infected cells. Following 48 h of treatment, the cells were infected with HSV-1 (5 × 10^4^ PFU) for 16 h and lysed. Cell lysates were separated in a gel and transferred onto a membrane incubated with primary antibodies α-tubulin and HSV-1 ICP0.

The mechanism of Lip^DOPE^-siHSV antiviral effect was elucidated by analyzing ICP0 protein levels. Lip^DOPE^-siHSV treatment resulted in a significant dose-dependent reduction of ICP0 levels, in comparison to untreated infected cells and SCR-siRNA (negative controls). Empty liposomes exhibited a moderate and similar reduction in ICP0 expression levels at both concentrations examined (Figure 9). The semi-quantification of the bands is shown in Figure S13.

### 3.6. Intracellular Fate

**Figure 10:**
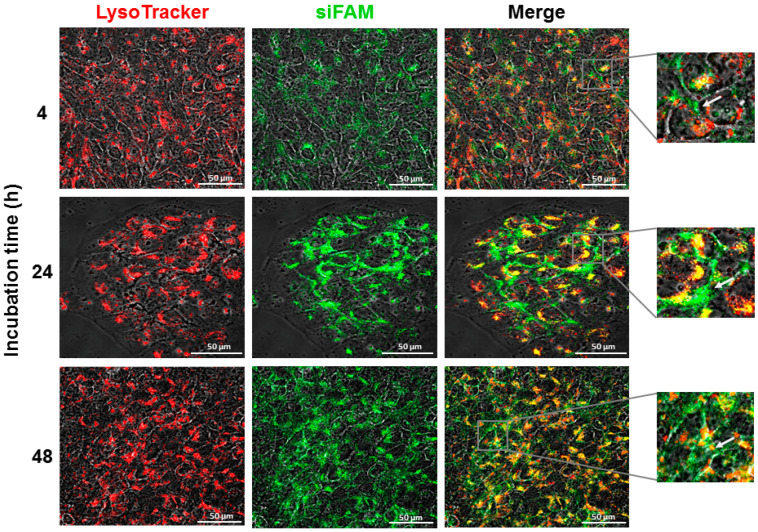
The time-dependent internalization (confocal microscopy) and intracellular fate of Lip^DOPE^-siRNA in keratinocytes (HaCaT) cell culture. Cells were treated with 6 µg/mL (siRNA concentration) and examined for siRNA endosomal/lysosomal escape. The endo-lysosomes are depicted in red (LysoTracker Red DND-99), fluorescently-labeled siRNA (siFAM) is depicted in green, and colocalization is depicted in yellow (white arrows). The fluorescence intensity was normalized to untreated cells; magnification ×60 (scale bar = 50 μm).

Treatment with liposomal siFAM (fluorescently labeled siRNA) revealed a partial release of the siRNA from the late endosomes/lysosomes of HaCaT cells after 4 h (Figure 10). Nevertheless, siRNA was substantially released from the endo-lysosomal compartments after 24 and 48 h, indicating a gradual endosomal escape to the cytoplasm (Figure 10; white arrows).

## 4. Discussion

Naked nucleic acids have a very short half-life due to rapid degradation by blood nucleases and extensive elimination by the liver and kidneys. Furthermore, cellular internalization is limited due to their both high negative charge and MW [54,55]. Therefore, successful siRNA delivery relies on a suitable delivery system [36,45,56]. Non-viral gene carriers are becoming a popular and safe alternative to current viral carriers, characterized by several advantages, including simplicity of manufacture, low immunogenicity, large payloads, flexibility of design, and multi-dose regimens [57,58,59,60]. Among them, liposomes are one of the most widely studied non-viral siRNA carriers due to their both biodegradability and biocompatibility [40,42,57,61,62]. In this study, we developed and examined a liposomal delivery system of siHSV and examined the role of various parameters, including formulation technique, lipid composition, and ratio (cationic lipids, ionizable cationic lipids, helper lipids, and PEGylated lipids). The developed siHSV liposomal formulations are characterized by unilamellar and spherical-shape vesicles, nanometer size range with a narrow PDI, PEGylated, and near-neutral surface charge for long residence time in the circulation [63]. The formulations were evaluated for cellular internalization and intracellular fate, in vitro and in vivo toxicity, biodistribution, and bioactivity, in cellular models of HSV-1 infection. It is suggested that the developed delivery system could serve pathologies associated with HSV-2 and VZV (varicella zoster virus) by encapsulating a suitable siRNA.

Liposome design requires a proper balance between the requirements of both stability and long circulation time and a facile siRNA release in the target cell. This necessitates appropriate selection of both cationic and helper lipids [40,53,62,64]. Encapsulation of the negatively-charged oligonucleotide sequences in lipidoic- or polymeric-based non-viral delivery systems is customarily achieved by complexation with a counter-ion [65,66]. DOTAP and DODMA, used in this study, are cationic lipids enabling the formation of complexes with the negatively-charged siRNA by electrostatic interaction, facilitating high siRNA loading [67]. DOTAP is a permanently ionized lipid (a quaternary ammonium), whereas DODMA is an ionizable cationic lipid, with a tertiary amine group, having a moderate pKa value. Its positive charge at low pH enables complexation with nucleic acids on one hand [67] and endosomal escape on the other. In addition, DOPE was introduced as an endosomal escape enhancer [53,68], and DSPC and cholesterol were included in the formulations as helper lipids providing membrane stability and intracellular delivery [52,53]. Large and/or charged particles are prone to rapid clearance by the mononuclear phagocytic system (MPS) [61,69,70,71]. In addition, the cellular uptake of negatively-charged vesicles is reduced due to electrostatic repulsion of the cell’s membrane, and positively-charged vesicles are associated with relatively low cellular uptake and hemostasis side-effects [72,73]. Consequently, the liposomes in this study were formulated with near-neutral charge (Table S1). Finally, DSPE-PEG was included in all liposomal formulation for increased residence time in the circulation. Surface shielding by PEGylation (e.g., DSPE-PEG) is a commonly used strategy for decreasing interactions between NPs and serum proteins [21], enhancing the NPs colloidal stability and circulation time [74]. However, it may decrease cellular internalization and hinder endosomal escape [53]; thus, a fine balance of membrane lipids composition was applied.

Lip^DOPE^-siHSV (N:P ratio of 5:1) was chosen for further studies since it is characterized with the highest siRNA loading (formulation #3; Table S1) and is expected to have increased propensity of releasing the siRNA due to the lower content of the complexing lipid. Reproducible batches have been obtained, which were found to be stable for a long period of time of 1.5 yr. at 4 °C (Figure 3a), as well as preserving both their size and siHSV integrity following serum incubation (Figure 3b and Figure 4).

Successful gene silencing using siRNA relies on effective cellular delivery [75]. Virtually all cells internalized the siHSV liposomes to a certain extent, reaching a maximum after 4 h (Lip^DOPE^-siHSV; Figure 5) or after 1 h (other formulations; Figures S3 and S4). Noteworthy, the internalization was time-dependent but not dose-dependent. Since HaCaT and SMC cell cultures are non-phagocytic, cellular internalization of liposomes depends on different endocytosis pathways, which involves four basic mechanisms: caveolae-mediated endocytosis, clathrin-mediated endocytosis, macropinocytosis, and clathrin- and caveolae-independent endocytosis [39,76,77].

Regardless of the specific internalization mechanism, the vesicles containing the nanoparticles fuse with sorting endosomes, which mature to late endosomes. In the late endosomes, the lipids and the cargo can either be released to the cytosol (lipids can be recycled back to the plasma membrane) or fuse with the lysosomes undergoing degradation [78]. Lysosomal degradation is a unique intracellular defense mechanism and one of the major challenges in drug-delivery systems. Liposomes and polymeric nanoparticles have been formulated for enabling endosomal escape via membrane fusion or membrane destabilization mechanisms, which have been shown as promising techniques to overcome this biological barrier [39,75,79,80,81]. Cationic charge is one of the most frequently proposed mechanisms for causing endosomal membrane destabilization. Persistent membrane destabilization can lead to pore formation in the endosomal membrane, resulting in leakage of molecules from the endosomes to the cytosol. Bursting of endosomes has also been proposed as a mechanism for endosomal escape, termed as the “proton sponge effect”. This is based on cationic polymers or lipids with excess uncharged protonable amine groups that can buffer endo-lysosomal acidification, leading to rupture [81]. The positively-charged lipid, DOTAP, could serve for endosomal escape. Nevertheless, it is suggested that the inclusion of DOPE in the best formulation, Lip^DOPE^-siHSV (formulation #3; Table 1 and Table S1), aided endosomal escape (Figure 10). It has been proposed that DOPE can facilitate the fusion of the nanocarrier with the endosomal membrane, facilitating escape of the cargo into the cytosol, with low cytotoxicity [68]. Thus, it is suggested that the beneficial effect of DOPE, promoting a non-lamellar lipid phase change by inflicting a conformational change upon acidification, was facilitated by the action of DOTAP, which destabilized the membrane, resulting in the effective release of siHSV to the cytosol [81].

Only one formulation of the six examined (formulation #4, Lip^DOD/DOT^; Table S1) was found significantly cytotoxic to HaCaT cells (Figure S5), and one formulation (#6, Lip^DOD3^; Table S1) exhibited moderate cytotoxicity (Figure S7). The observed cytotoxicity could be ascribed to the increased concentration of the counter-ions, DODMA and DOTAP, in formulation #4 (Table S1), and to the high concentration of DODMA in formulation #6 (Table S1). Cationic lipids, which interact with the membrane, could compromise the cell’s membrane integrity as well as the intracellular compartments leading to toxicity [82,83]. Indeed, the nontoxic formulations contained lower concentrations of the positively charged lipids exhibiting >70% viability (Figure 6, Figures S4 and S6).

Hepatotoxicity is a key concern in the clinical use of nanotherapeutics as preclinical studies have shown extensive accumulation in the liver [84,85]. It has been postulated that the reduced hepatotoxicity observed in clinical studies is due to the uptake of nanotherapeutics by macrophages in the liver [86]. No hepatotoxicity was detected following IV treatment of intact mice with Lip-siRNA (Figure S6b), which is promising for a potential therapeutic application.

Biodistribution studies of liposomal siRNA are important for evaluating target organ specificity and possible systemic toxicity. Gross pathology revealed no organ toxicity. The biodistribution studies in intact mice revealed that Lip^DOPE^ highly accumulated in visceral organs, especially the liver and spleen, which are known as the major disposal sites for particulate systems following systemic administration [87]. Lower accumulation was detected in the kidneys, lungs, heart, and CNS (brain, and spinal cord). Of note is the uptake by the trigeminal ganglion (Figure S14). Nevertheless, it is suggested that for enhanced efficacy, mediated by increased accumulation in the peripheral nervous system, a targeted delivery system would be required [59] and possibly conveyed by a topical application of the liposomal formulation embedded in a semi-solid preparation or local injection near the neural ganglia [88,89]; coated microneedles have been described for siRNA delivery to the skin [90].

The 2D plaque reduction assay of keratinocytes and the 3D epidermis model were used for assessing the antiviral effect of Lip-siHSV on the lytic virus. Lip^DOPE^-siHSV treatment resulted in the inhibition of viral plaques formation in HaCaT cultures in a dose-dependent-manner, whereas siHSV transfected by lipofectamine eradicated the virus at the lowest examined concentration exhibiting dose-independent antiviral efficacy (Figure 7). However, the latter is expected to be less efficient in vivo due to instability in serum as well as a significant degree of cytotoxicity and rapid clearance by the MPS that restricts its application in vivo [91,92,93,94,95,96,97,98,99]. Indeed, biodistribution studies revealed higher accumulation of Lip^DOPE^-siRNA in several organs in comparison to siRNA complexed with lipofectamine following IP administration in mice (Figure S15), most probably due to the better stability of siRNAs delivered by liposomes. Surprisingly, empty liposomes exhibited a moderate antiviral effect (Figure 7). The components of the formulation are well known as biocompatible, and, based on extensive experience with similar liposomal formulations, we did not find any biological effect of the components. Nevertheless, this apparent effect should be further examined in a broader dose-response study. In addition, since HSV-1 is an enveloped virus, it can be speculated that the overall lipid modification following liposomes internalization into epithelial cells might interfere with the ability of the virus to assemble into functional infectious virus particles. It is known that even under optimal in vitro conditions, only ~1% of the generated viral particles are functional [100]. Therefore, minor disturbances may cause a reduction in the virus’s proliferation and infection rates. It is noteworthy that, except for Lip^DOPE^-siHSV, all other developed siHSV formulations (Table S1) exhibited no antiviral effect (Figures S8–S10). It is suggested that the antiviral effect of Lip^DOPE^-siHSV, in contrast to all other formulations, is due to the observed endosomal escape of siHSV (Figure 10). All other liposomal siHSV formulations (e.g., Lip^DSPC^; Figure S11) were localized in the endo-lysosomal compartments, incapable of releasing the siRNA into the cytosol.

Plaque assays remain one of the most accurate methods for the quantification of infectious virions and of antiviral substances by counting the distinct plaques (infectious units and cellular dead zones) in cell culture [101,102]. However, it has been suggested that cells behave differently when they are grown in a 3D extra-cellular matrix involving cell interactions [103,104,105]. Although mouse models have been broadly utilized to study tissue morphogenesis in vivo, mouse and human skin have significant differences in cellular architecture and physiology, which makes it difficult to extrapolate mouse studies to humans [106,107]. The 3D skin model is a helpful strategy to bridge the gap between animal models and in vitro monolayer cells [108]. The HSV-infected 3D epidermis model is a multilayered stratified epidermis, which was generated from immortalized keratinocytes (Figure 8). In this model, Lip^DOPE^-siHSV was found to efficiently protect the epidermis tissue, similar to free siHSV (lipofectamine) treatment, demonstrating gene silencing and an inhibition of the infection caused by the virus (Figure 8). Importantly, the antiviral effect was obtained with undamaged morphology of the epidermis tissue layers (Figure S12).

ICP0 plays a key role in HSV-1 lytic replication and reactivation from latency [14,109]. The mechanism of liposomal siHSV antiviral effect was verified by silencing of ICP0 expression levels in a dose-dependent manner (Figure 9 and Figure S13). The modest reduction of ICP0 levels exhibited by the empty liposomes is consistent with the antiviral effect found in the 2D and 3D models, which is most probably due to an unspecific effect.

## 5. Conclusions

We have identified an siRNA (siHSV) that knocks down gene expression of ICP0. Several PEGylated liposomal formulations encapsulating siHSV have been developed and examined. The optimal liposomal siHSV formulation (Lip^DOPE^) was characterized by desirable physiochemical properties, in terms of nano-size, low polydispersity index, neutral surface charge, high siHSV loading, spherical shape, stability in physiologic conditions in vitro, and long-term shelf-life stability. The liposomes exhibited profound internalization by HaCaT cells, a gradual endosomal escape, and no cytotoxicity at the effective concentration range, and accumulated mainly in visceral organs and, to some extent, in the trigeminal ganglion, with no detrimental effect on mice liver enzymes. Lip^DOPE^ demonstrated significant antiviral efficacy in both a plaque reduction assay and in a 3D epidermis models, corroborated by decreased ICP0 levels. It is suggested that targeted liposomes to peripheral neurons should be examined in future in vivo studies for enhanced efficacy.

## Figures and Tables

**Table 1:** The physicochemical properties of Lip^DOPE^-siHSV.

Lip^DOPE^ DOPE:DOTAP:Cholesterol:DSPE-PEG, 1:1:2:0.3 (Molar Ratio)						
	N:P Ratio	Size (nm)	PDI	ζ Potential (mV)	siHSV Conc. (µg/mL)	Encapsulation Efficiency (%)
**siHSV**	16:1	142 ± 1	0.21 ± 0.03	2.7 ± 0.1	214 ± 25	86 ± 10
	5:1	133 ± 2	0.19 ± 0.02	3.3 ± 0.2	670 ± 36	89 ± 7
**Empty**	-	146 ± 2	0.24 ± 0.01	2.6 ± 0.6	-	-

Abbreviations: N/P ratio, amine:phosphate (cationic phospholipid and anionic siRNA, respectively); PDI, polydispersity index.

## Data Availability

Raw data are available upon request.

## Supplementary Materials

The following supporting information can be downloaded at: https://www.mdpi.com/article/10.3390/pharmaceutics14030633/s1, a pdf file. Figure S1: Genomic location of siRNA sequences on HSV1-Genome, Table S1: The physicochemical properties of liposomal formulations Figure S2: Cryo-TEM images of siHSV liposomal formulation, Figure S3: The internalization of Lip^DSPC^ by keratinocytes (HaCaT), Figure S4: The internalization and cytotoxicity of Lip^DSPE^ in keratinocytes (HaCaT), Figure S5: The internalization and cytotoxicity of Lip^DOD/DOT^ in keratinocytes (HaCaT), Figure S6: The in vitro and in vivo toxicity of Lip^DSPC^-siRNA, Figure S7: The cytotoxicity of Lip^DOD2^- and Lip^DOD3^-siHSV in keratinocytes (HaCaT), Figure S8: The antiviral effect of Lip^DSPC^-siHSV in the plaque reduction assay, Figure S9: The antiviral effect of Lip^DSPE^-siHSV in the plaque reduction assay, Figure S10: The antiviral effect of Lip^DOD^-siHSV in the plaque reduction assay, Figure S11: Internalization and intracellular fate of Lip^DSPC^-siRNA in keratinocytes (HaCaT), Figure S12: Representative H&E-stained sections in the 3D epidermis model, Table S2: Quantification of the infected area in the 3D epidermis model, Table S3: Quantification statistics, Figure S13: Quantification of ICP0 expression levels, Figure S14: The biodistribution of Lip^DOPE^ in mice, Figure S15: The biodistribution of Lip^DOPE^-siRNA in comparison to siRNA complexed with lipofectamine in mice.
