# Supplementary material for: Liposomal siRNA Formulations for the Treatment of Herpes Simplex Virus-1: In Vitro Characterization of Physicochemical Properties and Activity, and In Vivo Biodistribution and Toxicity Studies
Source: Pharmaceutics. 2022 Mar 13;14(3):633. doi: 10.3390/pharmaceutics14030633 (PMC8948811; doi:10.3390/pharmaceutics14030633)
Supplement: Supplementary file 1 [file pharmaceutics-14-00633-s001.zip › pharmaceutics-1613188-supplementary.pdf]

Supplementary Data

Liposomal siRNA formulations for the treatment of herpes simplex virus-1: *In vitro* characterization of physicochemical properties and activity, and *in vivo* biodistribution and toxicity studies

Doaa Jbara-Agbaria, Saskia Blondzik, Anke Burger-Kentischer, Majd Agbaria, Mirjam M. Nordling-David, Anna Giterman, Gil Aizik, Steffen Rupp and Gershon Golomb

1.	**Figure S1.**	Genomic location of siRNA sequences on HSV1-Genome.
2.	**Table S1.**	The physicochemical properties of liposomal formulations.
	**Figure S2.**	Cryo-TEM images of siHSV liposomal formulations.
3.	**Figure S3**	The internalization of Lip^{DSPC} by keratinocytes (HaCaT).
4.	**Figure S4.**	The internalization and cytotoxicity of Lip^{DSPE} in keratinocytes (HaCaT).
5.	**Figure S5.**	The internalization and cytotoxicity of $Lip^{DOD/DOT}$ in keratinocytes (HaCaT).
6.	**Figure S6.**	The *in vitro* and *in vivo* toxicity of Lip^{DSPC}-siRNA.
7.	**Figure S7.**	The cytotoxicity of Lip^{DOD2}- and Lip^{DOD3}-siHSV in keratinocytes (HaCaT).
8.	**Figure S8.**	The antiviral effect of Lip^{DSPC}-siHSV in the plaque reduction assay.
9.	**Figure S9.**	The antiviral effect of Lip^{DSPE}-siHSV in the plaque reduction assay.
10.	**Figure S10.**	The antiviral effect of Lip^{DOD}-siHSV in the plaque reduction assay.
11.	**Figure S11.**	Internalization and intracellular fate of Lip^{DSPC}-siRNA in keratinocytes (HaCaT).
12.	**Figure S12.**	Representative H&E-stained sections in the 3D epidermis model.
13.	**Table S2.**	Quantification of the infected area in the 3D epidermis model.
	**Table S3.**	Quantification statistics.
14.	**Figure S13.**	Quantification of ICP0 expression levels.
15.	**Figure S14.**	The biodistribution of Lip^{DOPE} in mice.
	**Figure S15.**	The biodistribution of Lip^{DOPE}-siRNA in comparison to siRNA complexed with lipofectamine in mice

1. siRNAs location in the viral genome.

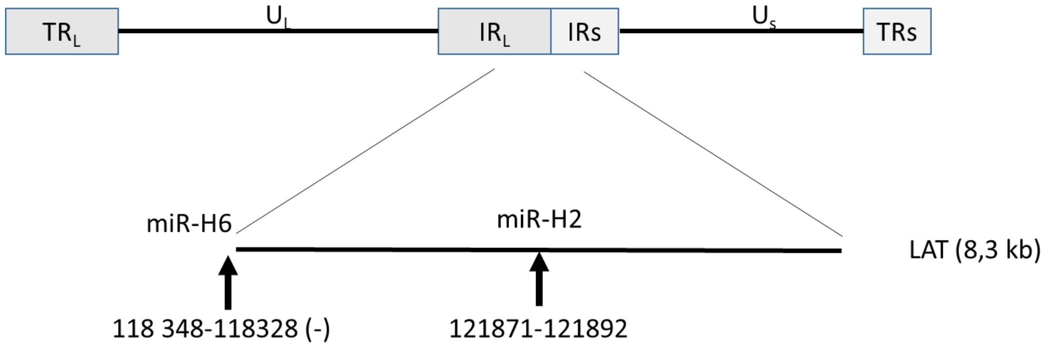

Figure S1. Genomic location of siRNA sequences on HSV1-Genome (adaped from Umbach et al. [8]). Schematic of the HSV-1 genome expanded to display the region of the LAT locus. LAT functions as a primary microRNA (miRNA) precursor that encodes four distinct miRNAs in HSV-1 infected cells. Sequence coordinates of viral miRNAs are given according to the HSV-1 strain 17 syn+ genome (NC 001806). Viral miR-H2 is oriented in the same orientation as LAT, miR-H6 in the opposite direction. TR, terminal repeat; IR, internal repeat; U_L, unique long; U_S, unique short. miR-H2-3p, is a transcribed antisense to infected cell protein (ICP0). Periodically, within an individual neuron, this silencing may be reversed, and HSV-1 is reactivated resulting in a recurrent disease.

2. Table S1. The physicochemical properties of PEGylated siHSV liposomal formulations examined (Mean±SD)

Formulation	Cargo	Lipid composition	Molar ratio	N/P Ratio	Size (nm)	PDI (nm)	ζ Potential (mV)	siHSV conc. (μg/ml)	EE (%)
**1. Lip^{DSPC}**	siHSV	DOTAP:DSPC: Cholesterol:DSPE-PEG	1:3:2:0.3	26:1	112±6.9	0.15±0.02	-3.0±1.7	46±4.6	64±8
	Empty			-	108±7.0	0.15±0.02	-5.1±4.4	-	-
**2. Lip^{DSPE}**	siHSV	DOTAP:DSPE: Cholesterol:DSPE-PEG	1:1:2:0.3	16:1	125±15	0.14±0.01	-1.7±0.3	81±6.0	65±4
	Empty			-	107±5.0	0.16±0.04	-2.0±0.3	-	-
**3. Lip^{DOPE}**	siHSV	DOTAP:DOPE: Cholesterol:DSPE-PEG	1:1:2:0.3	16:1	142±1.0	0.21±0.03	2.7±0.1	214±25	86±10
				**5:1**	133±2.0	0.19±0.02	3.3±0.2	670±36	89±7
	Empty			-	146±2.0	0.24±0.01	2.6±0.6	-	-
**4. Lip$^{DOD/DOT}$**	siHSV	DOTAP:DODMA:DOPC: Cholesterol:DSPE-PEG	3:5:7:4:0.8	16:1	144±0.2	0.20±0.01	4.7±0.3	180±4.3	72±6
				5:1	166±2.0	0.25±0.02	4.1±0.5	123±6.8	25±5
	Empty			-	141±0.2	0.11±0.01	3.8±0.3	-	-
**5. Lip^{DOD2}**	siHSV	DODMA:DSPC: Cholesterol:DSPE-PEG	5:1:4:0.16	10:1	183±0.5	0.18±0.02	-6.7±0.5	423±4.9	44±3
	Empty				184±1.0	0.19±0.01	-5.6±0.3	--	--
**6. Lip^{DOD3}**	siHSV		7.5:1:4:0.16	10:1	194±2.6	0.25±0.03	-6.5±0.1	351±5.2	36±7
	Empty			-	180±0.3	0.19±0.01	-7.4±0.6	--	--

Abbreviations: EE, Encapsulation efficiency; N/P ratio, amine (cationic phospholipid):phosphate (anionic siRNA)

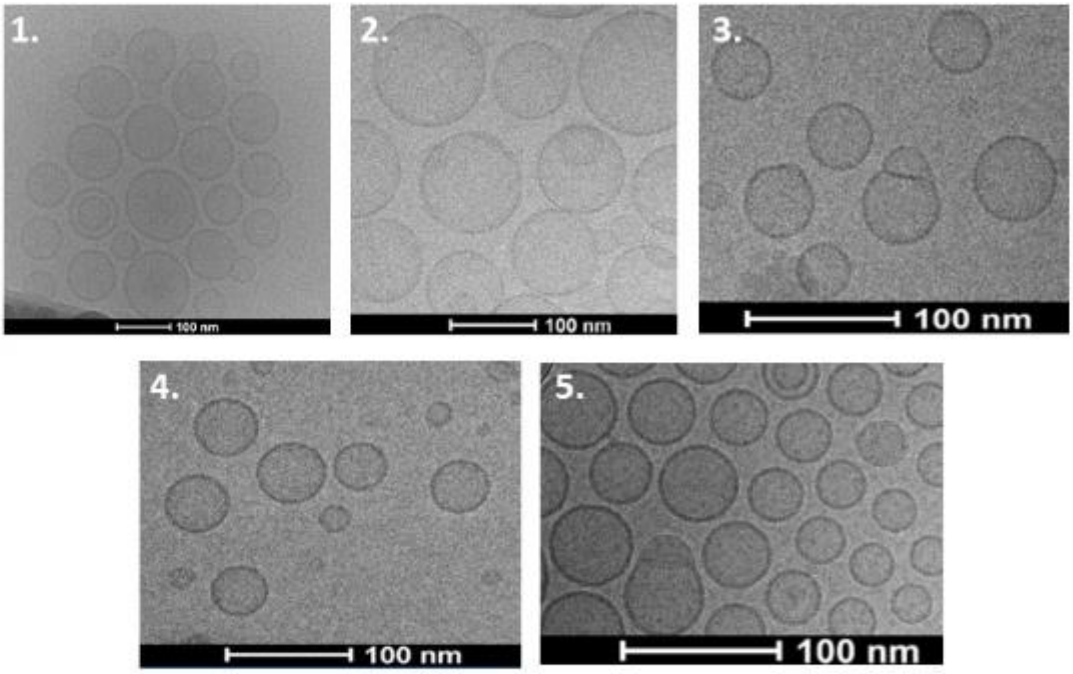

Figure S2. Representative cryo-TEM images depicting the structure of different siHSV liposomal formulations (scale bar=100 nm). The pictures are numbered as in Table S1.

3. The internalization of Lip^{DSPC} by keratinocytes (HaCaT).

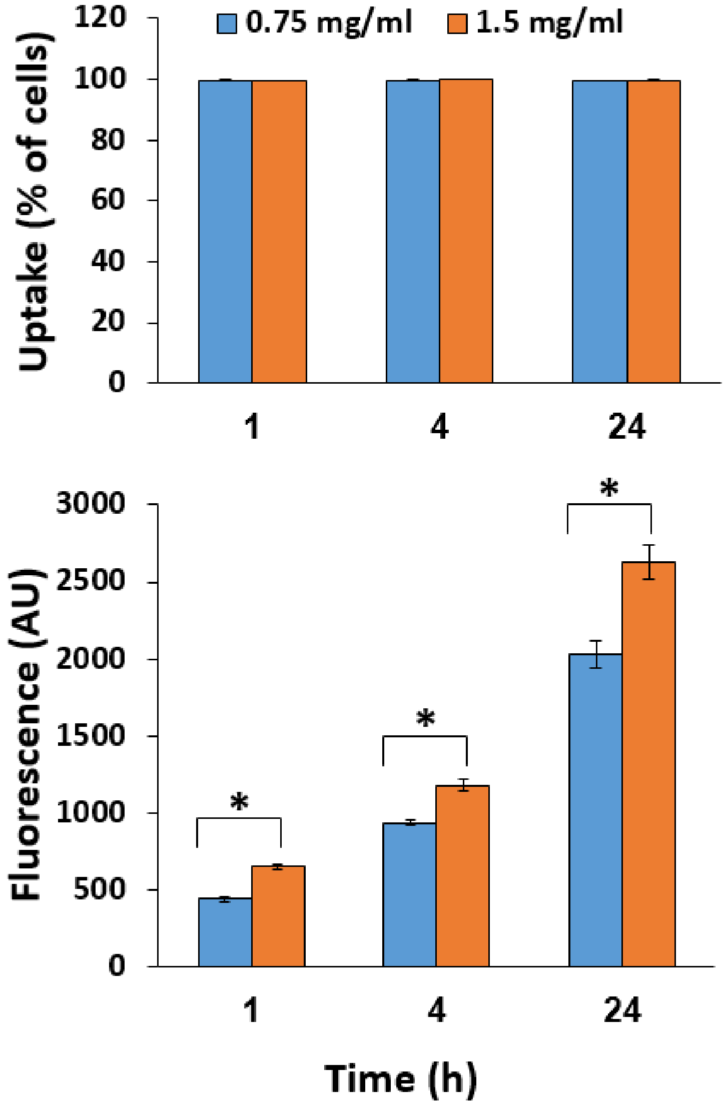

Fig. S3. Internalization over time of Lip^{DSPC} (2 mg/ml lipids; fluorescently labeled with Rhodamine-PE) in keratinocytes (HaCaT) cell culture, determined by FACS analysis. The fluorescence intensities were normalized to untreated cells (n=4; *$p<0.05$).

4. The internalization and cytotoxicity of Lip^{DSPE} in keratinocytes (HaCaT).

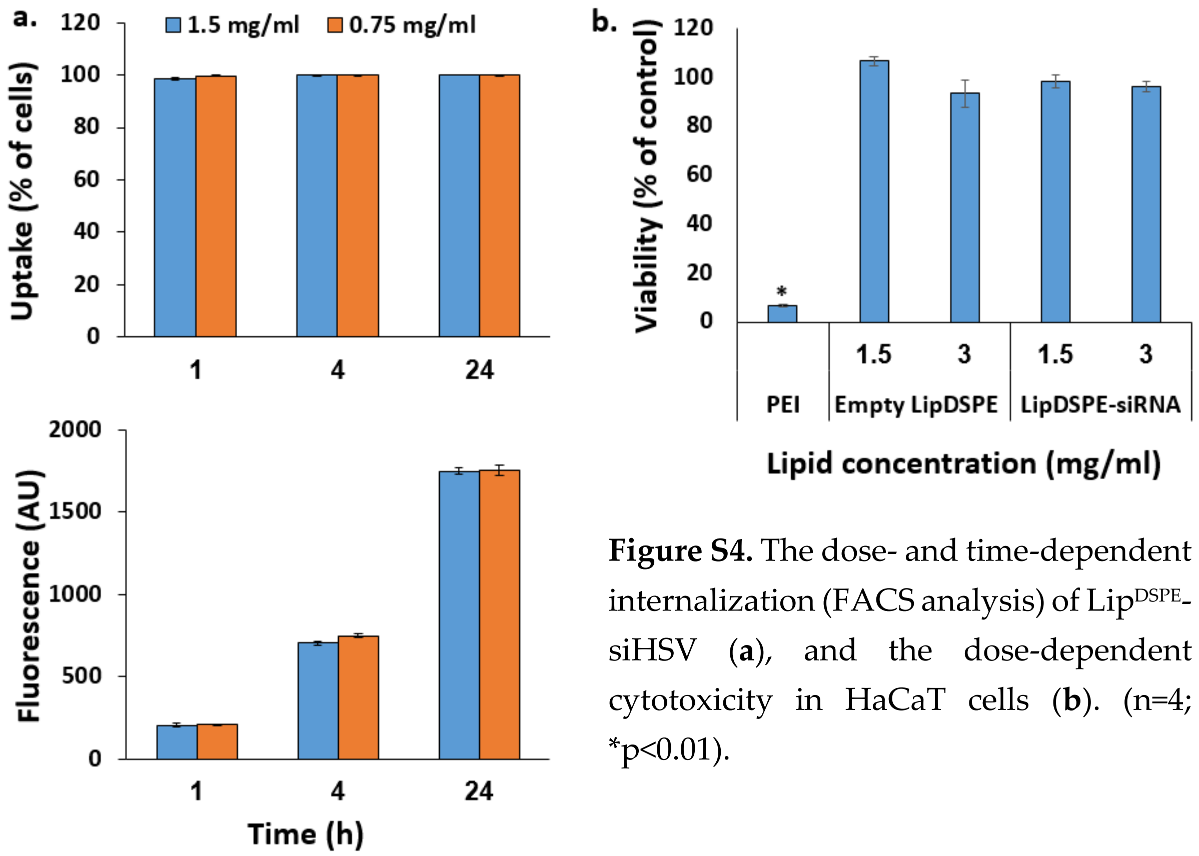

Figure S4. The dose- and time-dependent internalization (FACS analysis) of Lip^{DSPE}-siHSV (**a**), and the dose-dependent cytotoxicity in HaCaT cells (**b**). (n=4; *p<0.01).

5. **The internalization and cytotoxicity of Lip$^{DOD/DOT}$ in keratinocytes (HaCaT).**

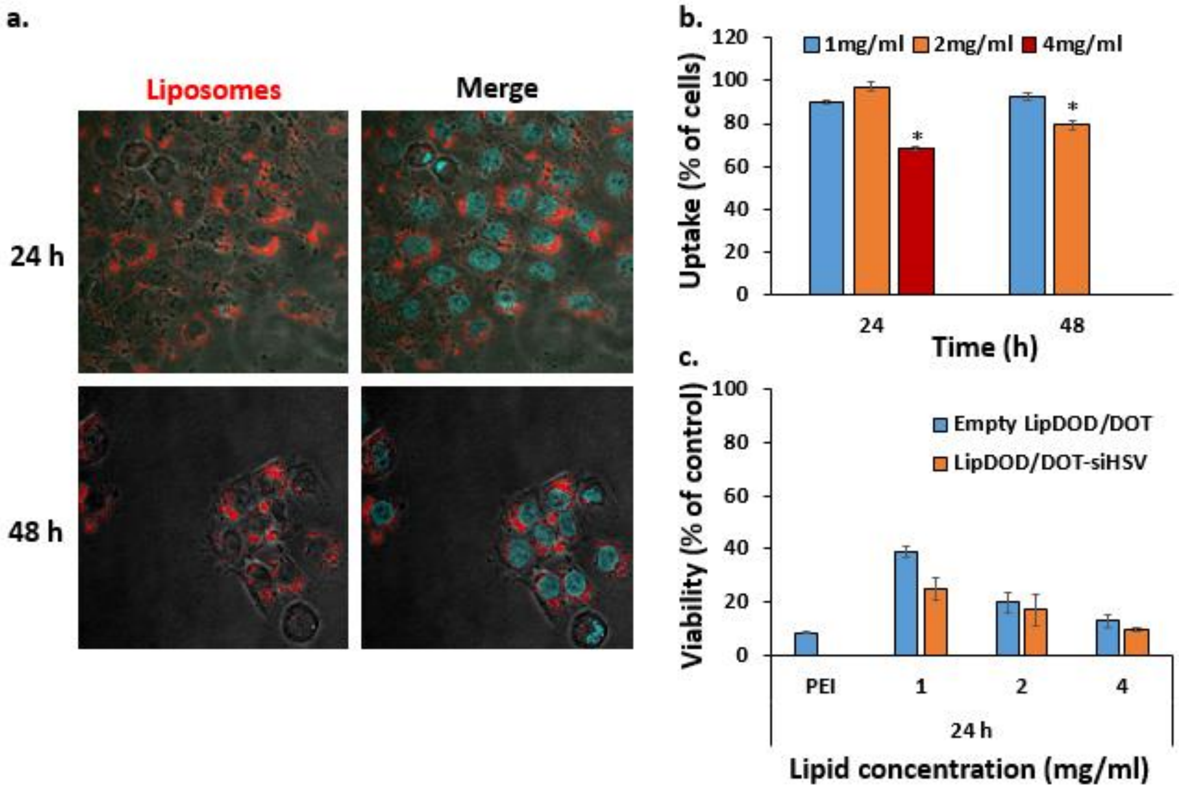

Figure S5. The qualitative (confocal microscopy; **a.**) and quantitative (FACS analysis; **b.**) internalization of fluorescently labeled Lip$^{DOD/DOT}$ (Rhodamine PE; 2 mg/ml lipids) by keratinocytes (HaCaT). The liposomal membrane is depicted in red, and cell's nuclei in cyan. The fluorescence intensities were normalized to untreated cells (n=4; *p<0.05). The dose-dependent cytotoxicity in keratinocytes cell culture (HaCaT) is shown in (**c**).

6. The *in vitro* and *in vivo* toxicity of Lip^{DSPC}-siRNA.

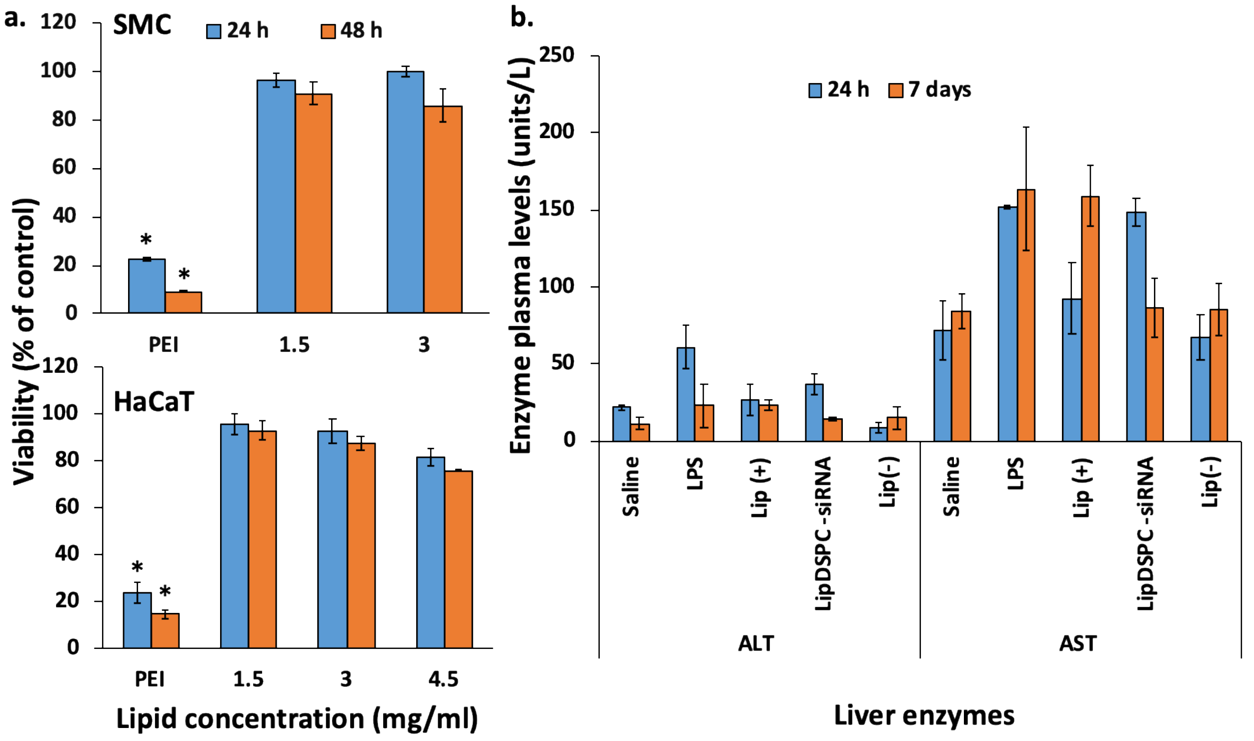

Figure S6. Evaluation of the *in vitro* and *in vivo* toxicity of liposomal siRNA (Lip^{DSPC}-siRNA). The dose- and time-dependent cytotoxicity of Lip^{DSPC}-siHSV in SMC and keratinocytes (HaCaT) are shown in (**a**). The cytotoxicity was determined by means of the MTT assay, and the cells viability was normalized to untreated cells (n=4; *p<0.01). **b**) The hepatotoxicity in mice following IV administration (2 µg/kg of siOPN [55] and 212 mg/kg lipids; n=8) determined by plasma levels of liver enzymes, alanine transaminase (ALT) and aspartate aminotransferase (AST), in comparison to positively- and negatively-charged liposomes. Lipopolysaccharide (LPS) treatment (IP) served as a positive control.

7. The cytotoxicity of Lip^{DOD2}- and Lip^{DOD3}-siHSV in keratinocytes (HaCaT).

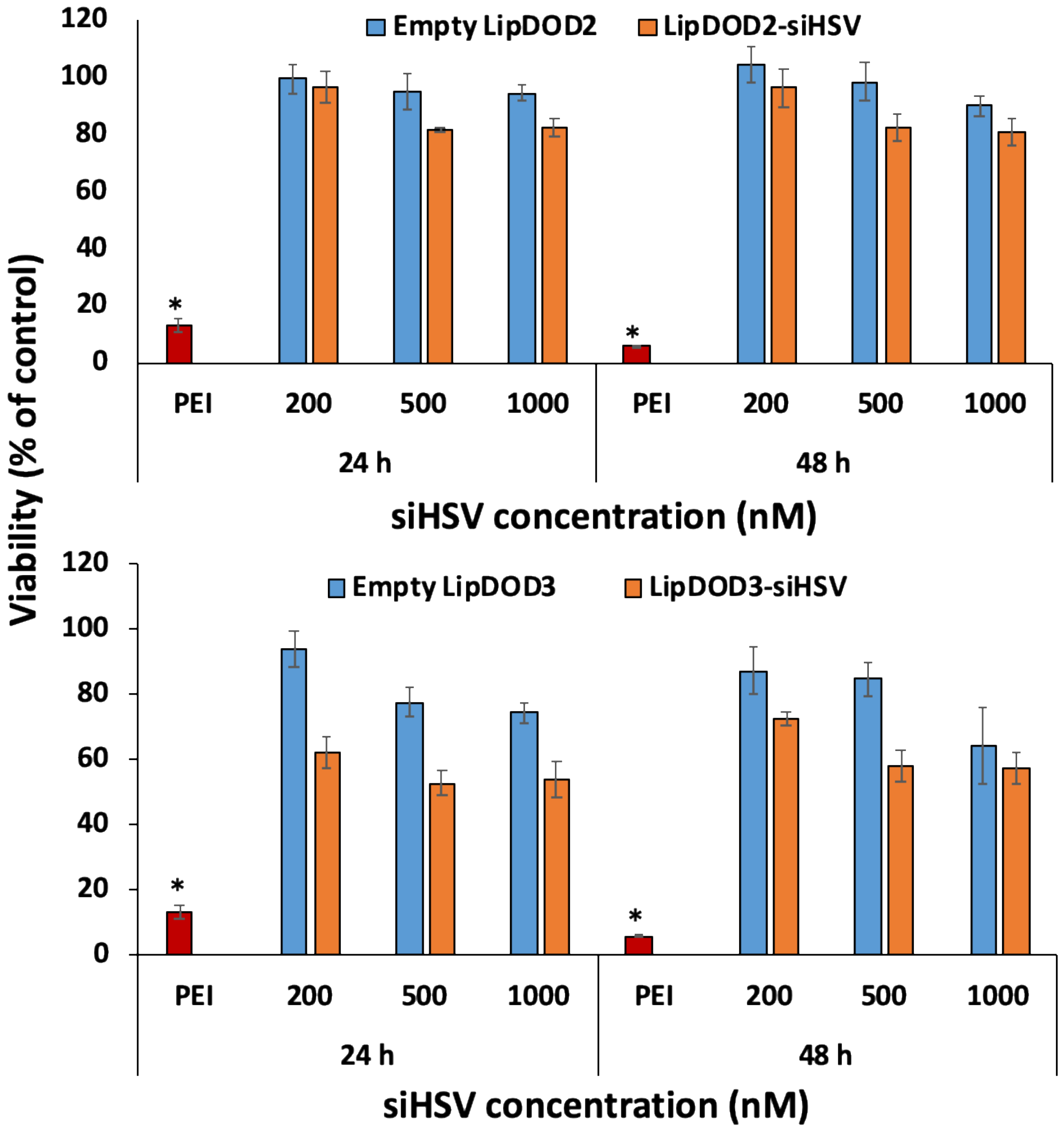

Figure S7. The dose- and time-dependent cytotoxicity of Lip^{DOD2}-siHSV and Lip^{DOD3}-siHSV in HaCaT cells. The cytotoxicity was determined by means of the MTT assay, and the cells' viability was normalized to untreated cells (n=4; *p<0.01).

8. The antiviral effect of of Lip^{DSPC}-siHSV in the plaque reduction assay.

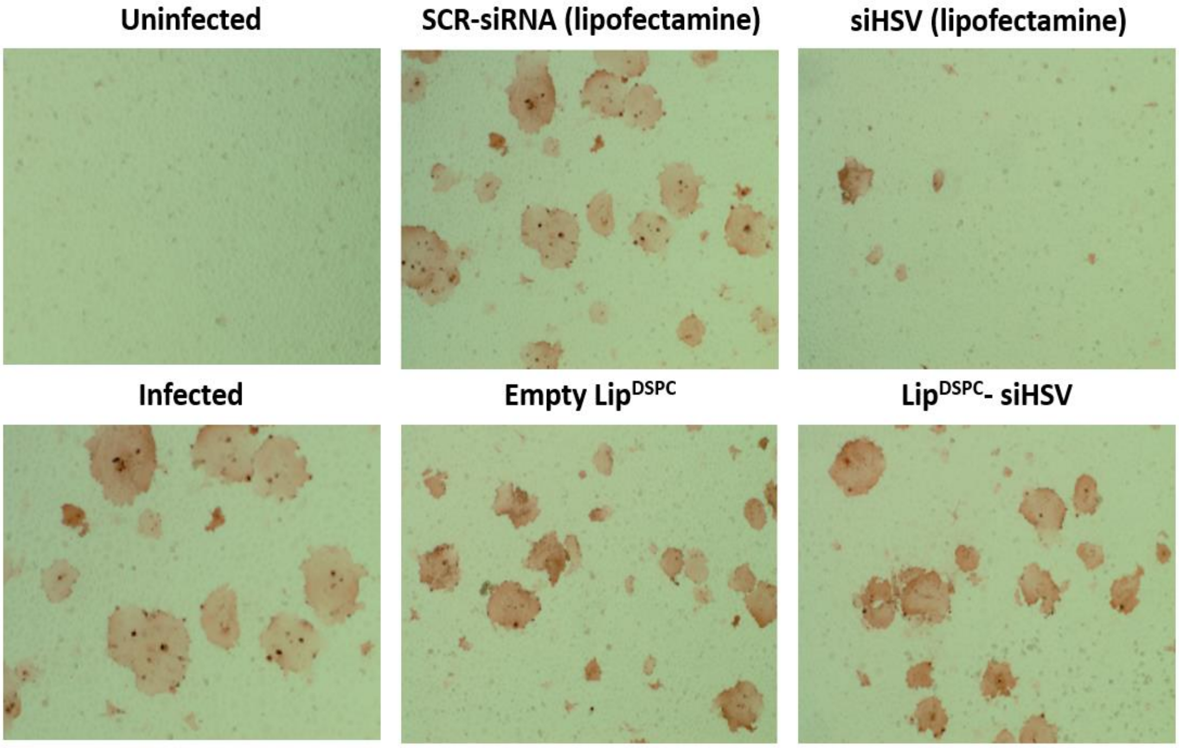

Figure S8. The antiviral effect of Lip^{DSPC}-siHSV (200 nM) following pre-treatment of HaCaT cells (keratinocytes) infected with HSV-1 (plaque reduction assay). Treatment groups: Lip^{DSPC}-siHSV, empty Lip^{DSPC} and scrambled-siRNA (SCR) transfected by lipofectamine (negative controls), and siHSV transfected by lipofectamine (positive control), in comparison to uninfected cells and untreated infected cells. Following 48 h of pre-treatment, the cells were infected with HSV-1 ($1x10^4$ PFU) for 16 h. The formed syncytia, shown in brownish red, was visualized following immunohistochemistry staining of the cells (anti-HSV1 antibody; n=2). Brownish red staining depicts the envelope-protein of the virus indicating proliferative infection; and no staining indicates full protection (magnification x10).

9. The antiviral effect of Lip^{DSPE}-siHSV in the plaque reduction assay.

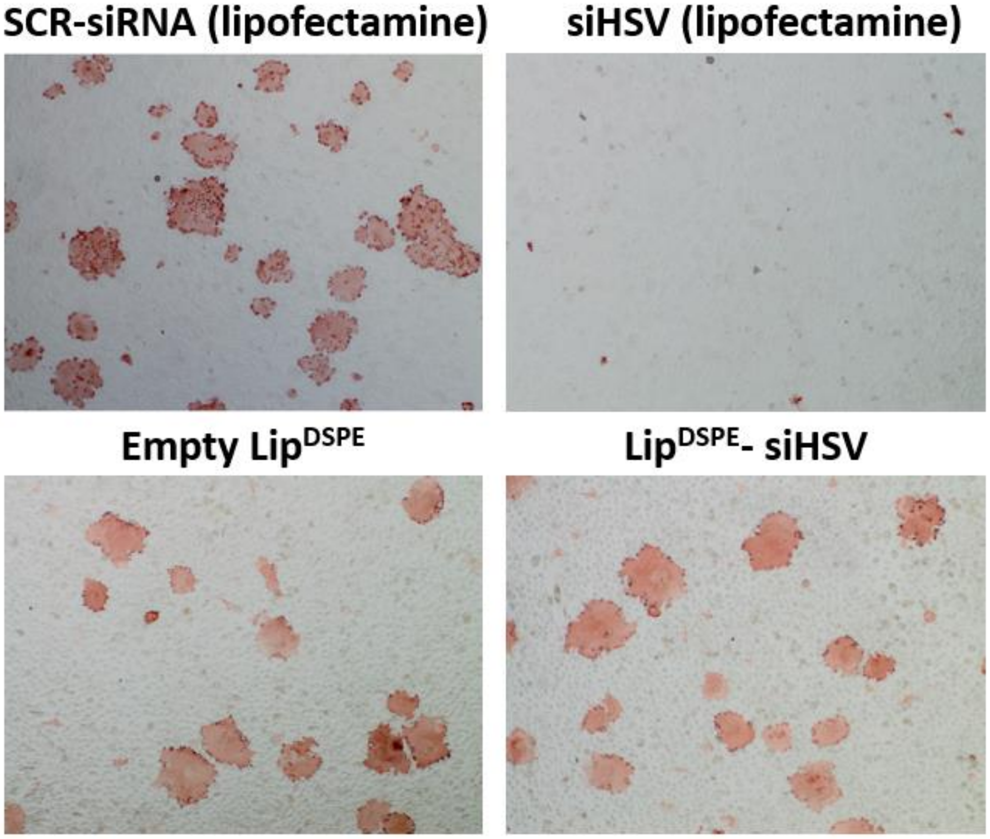

Figure S9. The antiviral effect of Lip^{DSPE}-siHSV (200 nM) following pre-treatment of HaCaT cells (keratinocytes) infected with HSV-1 (plaque reduction assay). Treatment groups: Lip^{DSPE}-siHSV, empty Lip^{DSPE} and scrambled-siRNA (SCR) transfected by lipofectamine (negative controls), and siHSV transfected by lipofectamine (positive control). Following 48 h of pre-treatment, the cells were infected with HSV-1 ($1x10^4$ PFU) for 16 h. The formed syncytia, shown in brownish red, was visualized following immunohistochemistry staining of the cells (anti-HSV1 antibody; n=2). Brownish red staining depicts the envelope-protein of the virus indicating proliferative infection; and no staining indicates full protection (magnification x10).

10. The antiviral effect of Lip^{DOD}-siHSV in the plaque reduction assay.

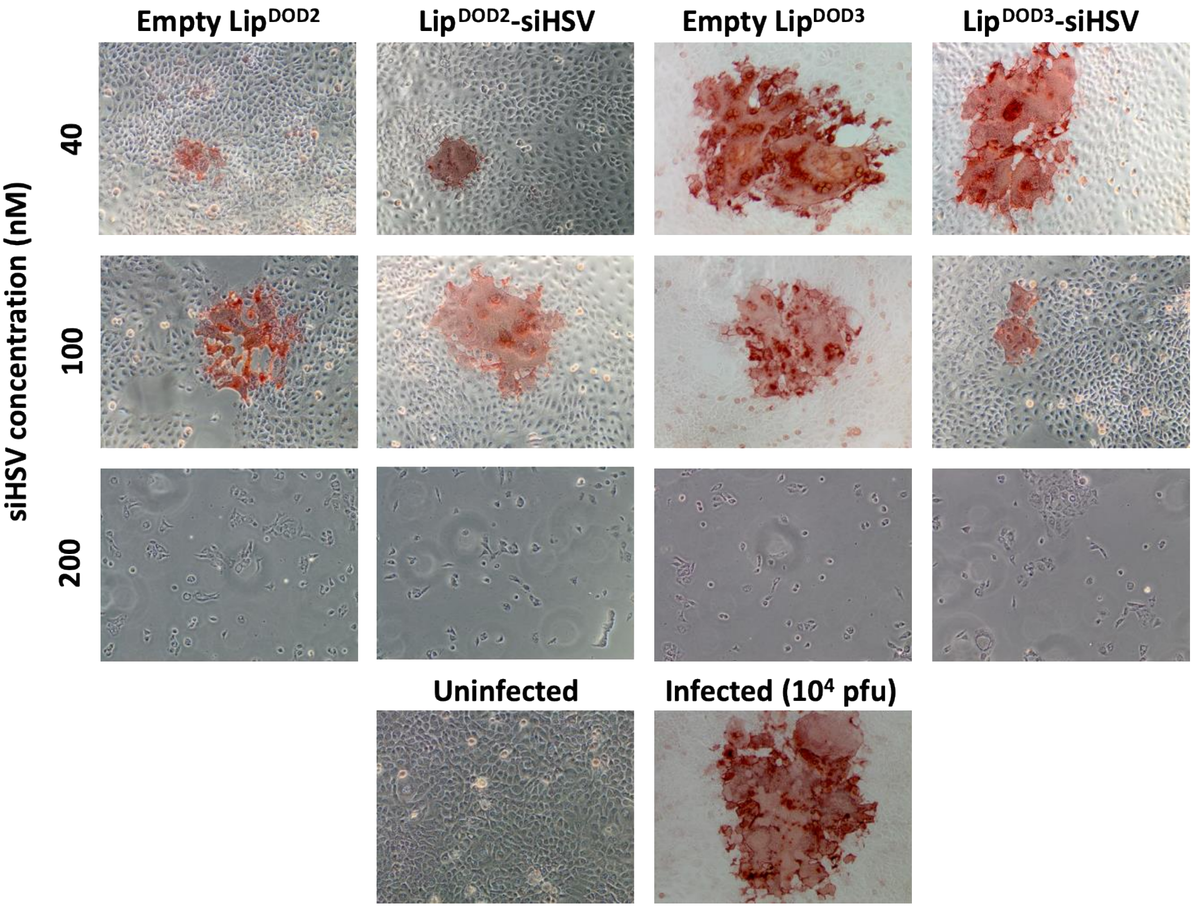

Figure S10. The antiviral effect of Lip^{DOD}-siHSV following pre-treatment of HaCaT cells (keratinocytes) infected with HSV-1 (plaque reduction assay). Treatment groups: Lip^{DOD2}-siHSV, Lip^{DOD3}-siHSV, and empty Lip^{DOD2} and empty Lip^{DOD3} (negative controls), in comparison to uninfected cells and untreated infected cells. Following 48 h of pre-treatment, the cells were infected with HSV-1 (1x10^4 PFU) for 16 h. The formed syncytia, shown in brownish red, was visualized following immunohistochemistry staining of the cells (anti-HSV1 antibody; n=2). Brownish red staining depicts the envelope-protein of the virus indicating proliferative infection; and no staining indicates full protection (magnification x10).

11. Internalization and intracellular fate of Lip^{DSPC}-siRNA in keratinocytes (HaCaT)

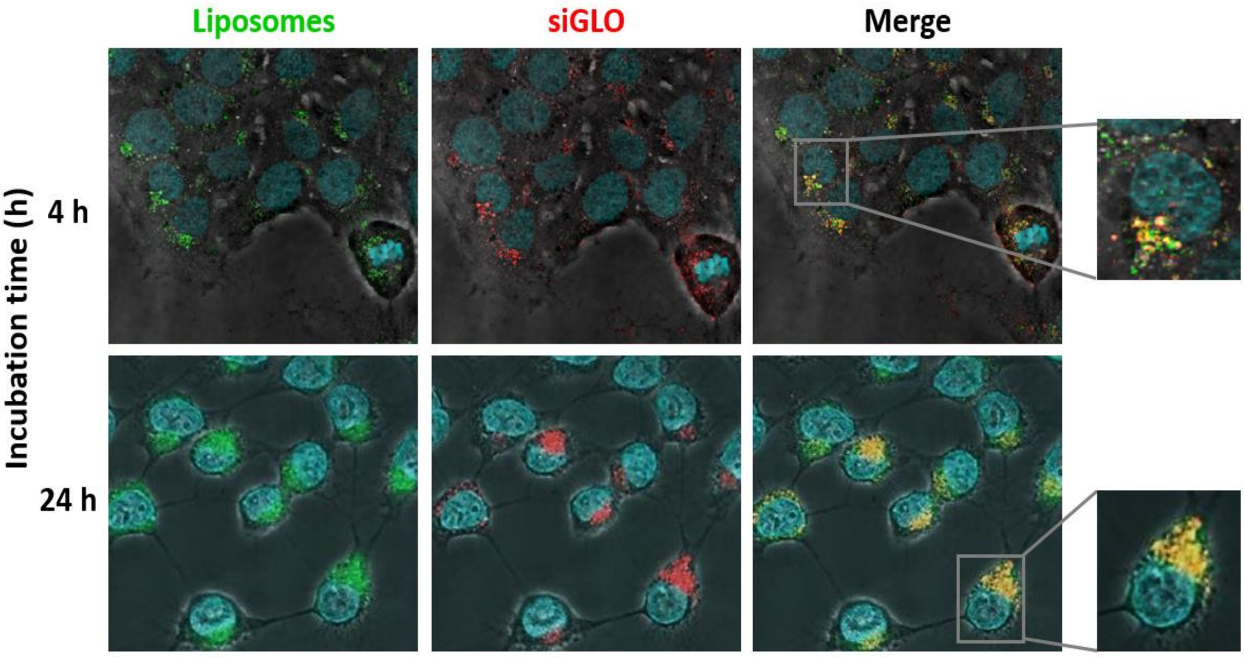

Figure S11. The time-dependent internalization and intracellular fate of Lip^{DSPC}-siRNA in keratinocytes (HaCaT) cell culture assessed by confocal microscopy. Cells were treated with Lip^{DSPC}-siGLO (double labeled liposomes, siGLO and fluorescein conjugated to DSPE (PE-CF); 1.5 mg/ml lipids), and were examined for siRNA endo-lysosomal escape. The liposomal membrane is depicted in green, the siRNA (siGLO) is depicted in red, and colocalization is depicted in yellow. The fluorescence intensity was normalized to untreated cells (magnification x60).

12. Representative H&E-stained sections of the epidermis in the 3D model.

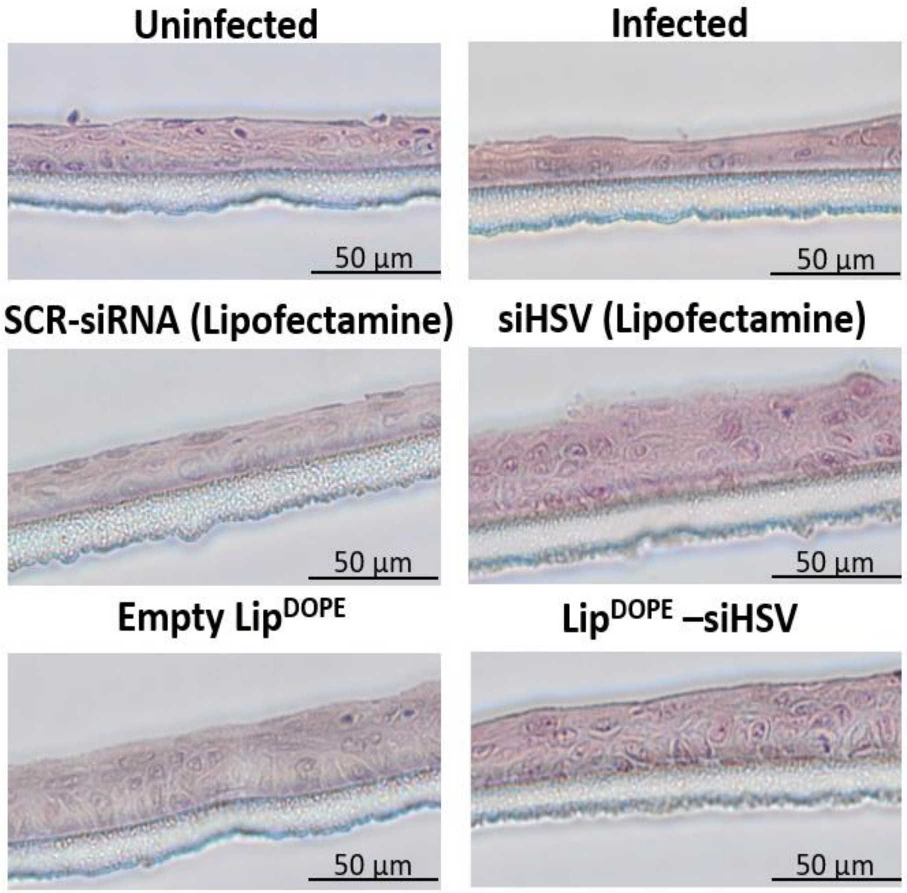

Figure S12. Representative H&E-stained sections of the epidermis in the 3D model depicting undamaged tissue by the virus (3 independent experiments with 2 or 3 individual skin models each; magnification x20).

13. The infected area in 3D epidermis models.

The infected area in the 3D epidermis model (Fig. 8) was quantified by means of ImageJ analysis (3 independent experiments in triplicates).

Table S2. Quantification of the infected area (A.U.) depicted in the epidermis tissue (Fig. 8).

Epidermis Model #	Uninfected	Infected	SCR-siRNA + Lipofectamine	siHSV + Lipofectamine	Empty Lip^{DOPE}	Lip^{DOPE}-siHSV N:P 5:1
1	0.001	0.255	0.607	0.038	0.021	0.077
	0.009	0.572	0.281	0.188	0.075	0.099
	0.002	0.334	0.356	0.167	0.202	0.182
2	0.002	0.122	0.046	0.024	0.097	0.057
	0.007	-	0.144	0.007	0.093	0.020
	0.007	0.211	0.065	0.021	0.099	0.084
3	0.003	0.281	0.201	0.067	0.040	0.018
	0.004	0.272	0.066	0.006	0.050	0.011
	0.002	-	0.055	0.035	0.014	0.016

Table S3. Quantification statistics.

	N	Mean	SD	Median
**Uninfected**	9	0.00411	0.00285	0.003
**Infected**	7	0.29243	0.13995	0.272
**SCR-siRNA + Lipofectamine**	9	0.20233	0.18727	0.144
**siHSV + Lipofectamine**	9	0.06144	0.06848	0.035
**Empty Lip^{DOPE}**	9	0.07678	0.05698	0.075
**Lip^{DOPE}-siHSV; N:P 5:1**	9	0.06267	0.05581	0.057

14. Quantification of ICP0 expression levels.

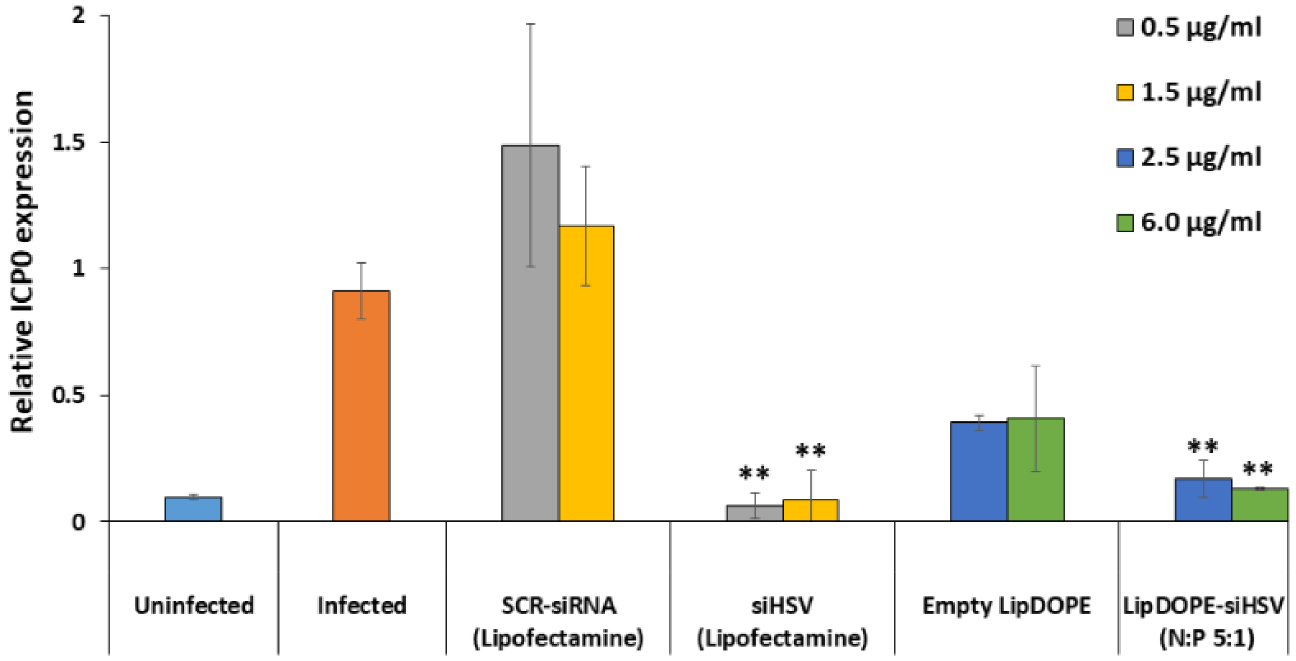

Figure S13. Quantification of ICP0 expression levels in the western blots (Fig. 9). The obtained ICP0 bands were semi-quantified by means of the ImageJ software and normalized to the expression of the reference gene, α-tubulin.

15. The biodistribution of Lip^{DOPE} in mice.

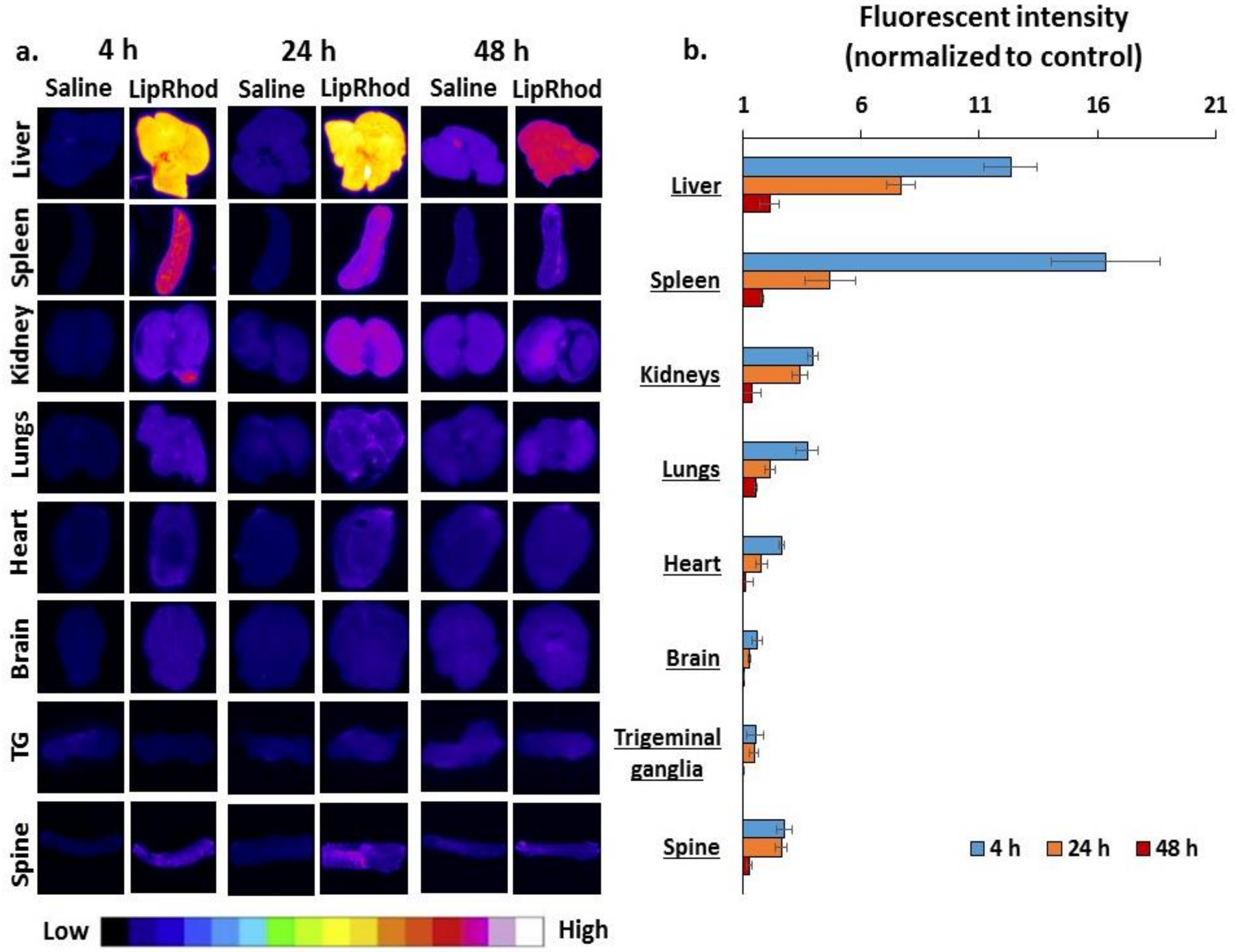

Figure S14. The biodistribution of Lip^{DOPE} (fluorescently labeled with Rhodamine-PE; LipRhod) in mice following IP injection (200µl of liposomes suspension (36mg/ml); Liss Rhod PE 0.125mg/ml). Selected organs (liver, spleen, kidneys, lungs, heart, brain, trigeminal ganglia and spine) were examined by means of a Typhoon scanner (**a**), followed by ImageJ software analysis (**b**). The fluorescence intensities were normalized to saline-treated animals (mean ± SD; n=4 at each time point).

The biodistribution of Lip^{DOPE}-siRNA in comparison to siRNA complexed with lipofectamine in mice

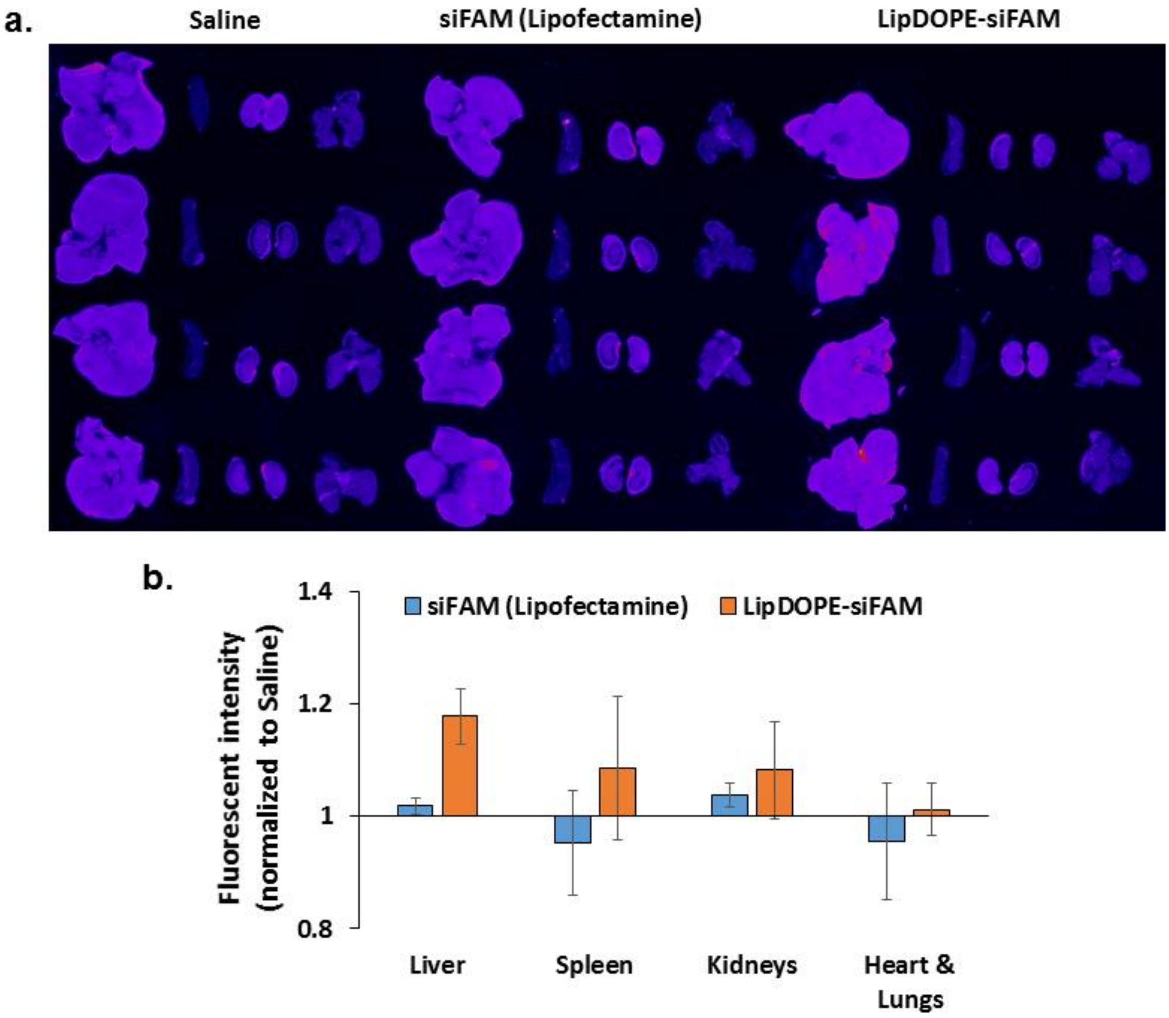

Figure S15. The biodistribution of Lip^{DOPE}-siRNA (fluorescently labeled with siFAM (5% of total siRNA) in comparison to siRNA complexed with lipofectamine in mice following IP injection (2mg/kg). Mice were euthanized 24 h following treatment and selected organs (liver, spleen, kidneys, heart and lungs) were examined by means of a Typhoon scanner followed by ImageJ software analysis. The fluorescence intensities were normalized to saline-treated animals (mean ± SD; n=4 at each group).
